# Satellitome analyses in nematodes illuminate complex species history and show conserved features in satellite DNAs

**DOI:** 10.1186/s12915-022-01460-7

**Published:** 2022-11-18

**Authors:** Evelin Despot-Slade, Saša Širca, Brankica Mravinac, Philippe Castagnone-Sereno, Miroslav Plohl, Nevenka Meštrović

**Affiliations:** 1 Division of Molecular Biology, Ruđer Bošković Institute, Bijenička cesta 54, 10000 Zagreb, Croatia; 2 Agricultural Institute of Slovenia, Ljubljana, Slovenia; 3 INRAE, CNRS, Université Côte d’Azur, ISA, 06903 Sophia Antipolis, France

**Keywords:** Repetitive DNA, Satellitomes, Phylogenomics, Conserved features, Satellite DNA transcriptome, Nematode

## Abstract

**Background:**

Satellite DNAs (satDNAs) are tandemly repeated non-coding DNA sequences that belong to the most abundant and the fastest evolving parts of the eukaryotic genome. A satellitome represents the collection of different satDNAs in a genome. Due to extreme diversity and methodological difficulties to characterize and compare satDNA collection in complex genomes, knowledge on their putative functional constraints and capacity to participate in genome evolution remains rather elusive. SatDNA transcripts have been detected in many species, however comparative studies of satDNA transcriptome between species are extremely rare.

**Results:**

We conducted a genome-wide survey and comparative analyses of satellitomes among different closely related *Meloidogyne* spp. nematodes. The evolutionary trends of satDNAs suggest that each round of proposed polyploidization in the evolutionary history is concomitant with the addition of a new set of satDNAs in the satellitome of any particular *Meloidogyne* species. Successive incorporation of new sets of satDNAs in the genome along the process of polyploidization supports multiple hybridization events as the main factor responsible for the formation of these species. Through comparative analyses of 83 distinct satDNAs, we found a CENP-B box-like sequence motif conserved among 11 divergent satDNAs (similarity ranges from 36 to 74%). We also found satDNAs that harbor a splice leader (SL) sequence which, in spite of overall divergence, shows conservation across species in two putative functional regions, the 25-nt SL exon and the Sm binding site. Intra- and interspecific comparative expression analyses of the complete satDNA set in the analyzed *Meloidogyne* species revealed transcription profiles including a subset of 14 actively transcribed satDNAs. Among those, 9 show active transcription in every species where they are found in the genome and throughout developmental stages.

**Conclusions:**

Our results demonstrate the feasibility and power of comparative analysis of the non-coding repetitive genome for elucidation of the origin of species with a complex history. Although satDNAs generally evolve extremely quickly, the comparative analyses of 83 satDNAs detected in the analyzed *Meloidogyne* species revealed conserved sequence features in some satDNAs suggesting sequence evolution under selective pressure. SatDNAs that are actively transcribed in related genomes and throughout nematode development support the view that their expression is not stochastic.

**Supplementary Information:**

The online version contains supplementary material available at 10.1186/s12915-022-01460-7.

## Background

Eukaryotic genomes exhibit high proportion of repetitive DNA sequences, including transposable elements (TEs) and satellite DNA (satDNAs). In contrast to interspersed TEs, satDNAs are arranged as long arrays of tandemly repeated units. They are considered as ones of the most abundant repeated sequences and among the fastest evolving parts of the eukaryotic genome. A satellitome represents the collection of different satDNAs in a genome [1]. Due to their repetitive nature, these abundant genome fractions remain the most poorly mapped in assembled genomes (reviewed in [2]). Consequently, an insight into genome-wide composition of satDNAs is still limited and knowledge of their capacity to participate in genome organization, function and evolution remains rather elusive. Due to extreme divergence of satDNAs and random homoplasy generated by the chance amplification of satDNAs, they have also been largely avoided as a phylogenetic marker [3].

SatDNA sequences evolve in a concerted manner, where accumulation of mutations follows homogenization within a genome and fixation within a species [4]. The result of this process is lower intra-specific than inter-specific sequence variability. However, concerted evolution is not the sole process which can explain the extremely dynamic evolution of satDNA sequences observed even in closely related species. The library model proposes the occurrence of dynamic satDNAs profile as a result of differential amplifications and/or contractions of satDNAs from a satDNA library [5, 6]. Recent study of satellitomes in two grasshoppers suggests satDNA evolution as the result of recursive cycles of amplification and degeneration which lead to contingent evolutionary pathways [7]. Persistence of satDNAs in related genomes during long evolutionary periods as well as appearance of conserved motif and/or differential variability along the repeat unit sequence found in satDNAs of various species [8–11] suggest selective constraints in formation of some satDNAs [12]. Although functional constraints might be the cause of the preservation of satDNAs, their functional role is largely difficult to be proved. Among all detected conserved regions, a function is only assigned to the CENP-B box of alpha satDNA in human, which is proposed to act as a centromeric CENP-B protein binding site [13, 14]. Anyway, it is indisputable that satDNAs represent the main structural component of almost all monocentric centromeres implying their importance in centromere determination (reviewed in [15]). Specifically, studies have suggested a satDNA role in chromatin packaging [16] and centromere formation/maintenance [17]. In contrast to conserved centromere function, comprehensive bioinformatic analyses of centromeric satDNAs in a number of animal and plant species confirmed the rapid evolution of satDNAs in these areas [18]. Consequently, it has been considered that their extreme sequence diversity may represent a major evolutionary force that could result in hybrid incompatibilities and thus has an important role in speciation processes [19, 20]. Recent studies of satDNAs expression in (peri)centromeric heterochromatin unraveled participation of satDNA transcripts in various cellular processes such as *de novo* heterochromatin formation in mammals (reviewed in [21]), kinetochore formation in *Drosophila* [22] and up-regulation of X-linked genes in *Drosophila* [23]. Intriguingly, new studies have also provided evidences for role of satDNA transcripts in the process of malignant transformation, thus indicating their impact in cancer progression [24, 25]. Although satDNA transcription has become a focus of interest in the recent years regarding its pathophysiologic contribution, our knowledge concerning significance of satDNAs transcripts in normal physiological conditions is still rather limited.

The root-knot nematodes (RKN) of the genus *Meloidogyne* comprises globally important plant parasites responsible to ∼5% of damages to world agriculture. Their reproductive modes range from sexual to obligate asexual reproduction. The most widespread and economically important are obligatory mitotic parthenogenetic species of the *M. incognita* group (MIG) which includes *M. incognita*, *M. arenaria*, and *M. javanica*. The MIG species are closely related, as it was confirmed by their mitochondrial genomes whose comparative phylogeny could not discriminate particular species [26]. These species have previously been suggested to be polyploids, and phylogenetic analysis of nuclear loci revealed co-existence of several versions of the nuclear markers in each species [27]. In general, polyploidy can arise as a result of genome duplication(s) within a species (autopolyploidy) or from hybridization of different closely related species (allopolyploidy). Recent comprehensive comparative analyses of coding genome parts of closely related MIG species hypothesized the additive interspecies hybridization as the main process in MIG species formation [28, 29]. Genome sizes of 189, 297 and 304 Mb were estimated for *M. incognita*, *M. javanica* and *M. arenaria*, respectively [28]. The genomes of these asexual *Meloidogyne* were ~3–5 times bigger than the haploid genome size of the sexual *M. hapla* which is in accordance with hypothesis about their polyploidization. The MIG species are highly variable with respect to their chromosomal complement. The chromosome number ranges from 30 to 50, and thus, they are thought to be either diploids or triploids [30]. Although haploid number of the *Meloidogyne* genus is *n* = 18, polyploidy species such as those from the MIG group rarely have an exact multiple of 18 chromosomes due to different structural rearrangements.

Concerning genome composition, transposable elements (TEs) covered even 50.0% of the MIG genome assemblies, comprising ~1.7 times higher proportion of the genomes compared to the sexual relative *M. hapla*. There is a hypothesis that high abundance of TEs in mitotic MIG species might participate in their plasticity [28]. Genome-wide analyses of the other repetitive portions of the genome, such as satDNAs, have not been carried out so far. However, the most abundant satDNAs have been characterized by classical methods in various *Meloidogyne* species. The data revealed different A+T rich satDNA families with 170–300 bp repeat units and abundance up to 20% of the genome, as found for *M. fallax* (e.g., [31, 32]). It has also been shown that they evolved according to the library concept [12]. In addition, the study of the satDNA library of the three related satDNAs differently amplified in *Meloidogyne* species indicates selection as a limiting factor in formation and persistence of satDNAs in the library [12]. The distribution profile of six different satDNAs, in terms of their presence/absence in related *Meloidogyne* genomes, has been shown to be informative about phylogenetic relationships of these species [33]. However, those studies were limited only to small subset dominant satDNAs, due to the lack of appropriate methodology at that time.

Here, we performed comprehensive satellitome study at the genome scale in four *Meloidogyne* species by using a bioinformatic analysis of NGS reads [34] in order to disclose evolutionary trends of whole satDNA complements in closely related genomes. Based on the comparative study of this non-coding genome part, our approach proved to be helpful to elucidate complex species history. Further, detection of numerous satDNAs common to *Meloidogyne* related genomes enabled comparative analyses of global satDNA transcriptional pattern across the different species and life stages. In addition, the sequence comparison of 83 satDNAs found in related *Meloidogyne* genomes made possible the identification of conserved sequences’ features.

## Results

### Comparative analysis of satellitomes

**Fig. 1:**
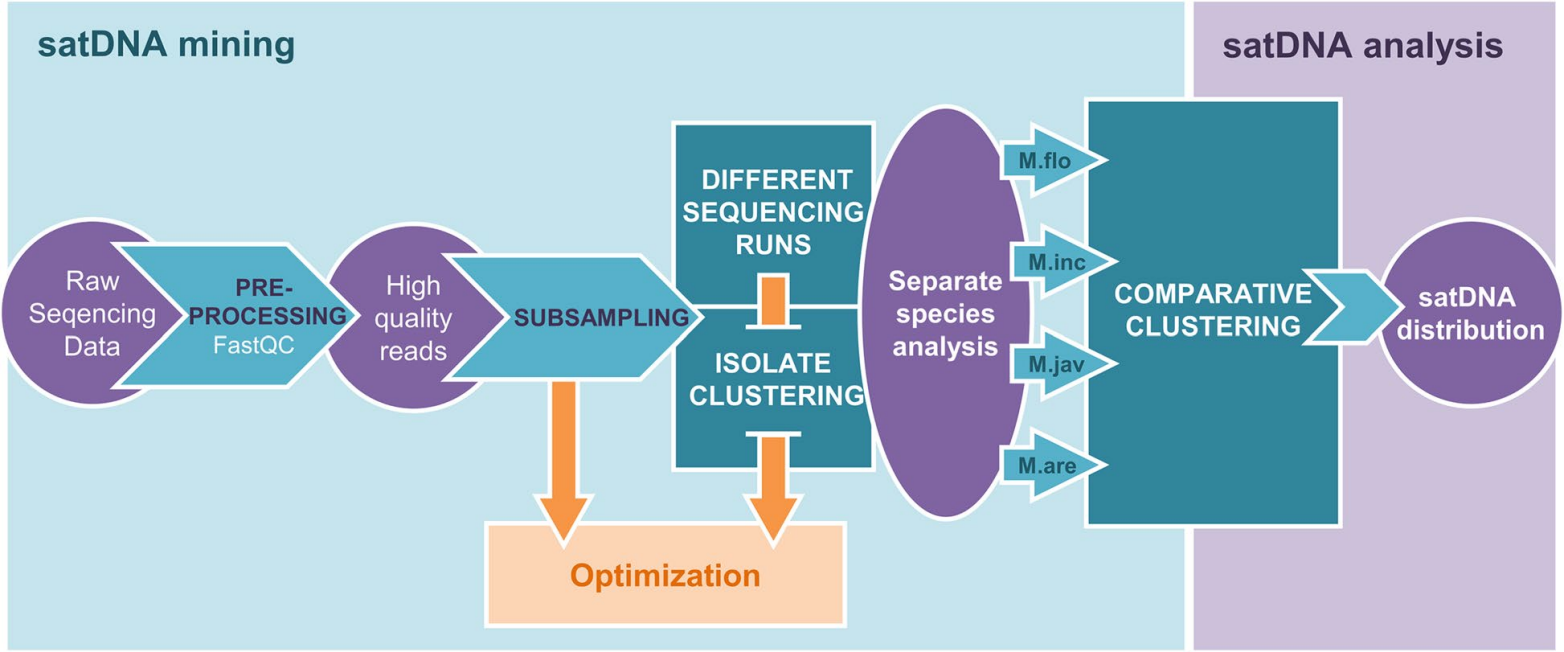
Workflow of satDNA mining in *Meloidogyne* species (M.flo-*M. floridensis*, M.inc-*M*. *incognita*, M.jav-*M. javanica* and M.are-*M. arenaria*) using graph-based clustering [34]. All major steps and obtained data are listed with more detailed description provided in Methods section

In order to characterize and compare satDNAs on the whole genome scale in four *Meloidogyne* species, *M. floridensis*, *M. incognita*, *M. arenaria*, and *M. javanica*, graph-based clustering of publicly available Illumina sequence reads [29] was performed using RepeatExplorer [34] (Fig. 1). This bioinformatic tool characterizes and quantifies the complete satDNA fraction of a genome using low-coverage sequencing reads and graph-based algorithm. To determine the optimal genome coverage which ensures accuracy of the satDNA identification, we used a genome coverage range from 0.125 to 0.5x in analyses for each *Meloidogyne* species (Additional file 1: Fig. S1). This genome coverage range has been proposed in previous comparative studies of satellitomes [35]. To provide equal sensitivity for all species, the number of analyzed reads was proportional to previously predicted genome sizes. Genome sizes of *M. incognita*, *M. javanica* and *M. arenaria* were estimated by flow cytometry [28], while *M. floridensis* genome size was estimated based on the assembled genome data [29]. The satellitome analyses with different genome coverages did not show strong deviations in the number of obtained satDNA clusters for *M. incognita*, *M. javanica* and *M. arenaria*, while the results for *M. floridensis* showed lower number of clusters in analyses with 0.125x coverage (Additional file 1: Fig. S1). To ensure high sensitivity of satDNA detection, genome coverage of 0.25x was selected for further comparative satellitome analyses in all species. In addition, to verify reproducibility of graph-based clustering at the species level, comparative analyses of satDNAs from different isolates were performed (Additional file 1: Fig. S2). The results show high qualitative and quantitative reproducibility between the three different isolates of *M. incognita* and *M. javanica* (Additional file 1: Fig. S2A and B) with difference in only one satDNA cluster between isolates of *M. javanica* and in three clusters in *M. incognita*. Based on these results, isolates M.inc-79 and M.jav-78 which have a maximal number of satDNA clusters were included in comparative satellitome analysis between species. In contrast, the analyses of three *M. arenaria* isolates show qualitative and quantitative variability (Additional file 1: Fig. S2C). Intra-specific variability in *M. arenaria* has also been detected in analyses of isozyme phenotypes and mitochondrial haplotypes [36] as well as in comparative analyses of coding regions [29]. To prevent bias in comparative species analyses due to *M. arenaria* intra-specific variability, reads which represent all three isolates of *M. arenaria* were included in analyses. Since only NGS data set for one isolate of *M. floridensis* was available, intra-specific analysis could not be implemented for *M. floridensis*.

**Fig. 2:**
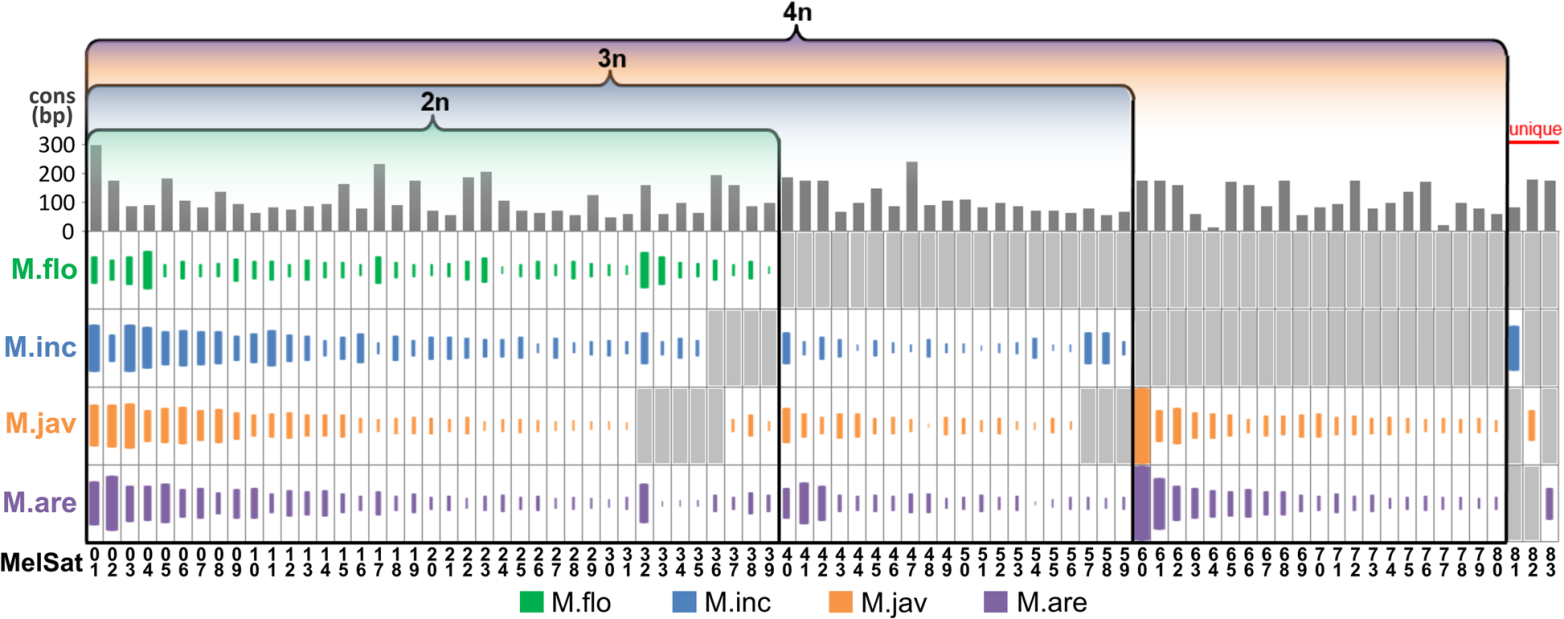
Comparative analyses of satellitomes of the four *Meloidogyne* species (M.flo-*M. floridensis*, M.inc-*M*. *incognita*, M.jav-*M. javanica* and M.are-*M. arenaria*) based on satDNAs clustering. The grey bar plots at the top show the length of satDNA repeat unit for each satDNA cluster (MelSat). The area of the colored rectangles is proportional to the abundance of individual satDNA in a corresponding genome (ranged from 0.002 to 0.613%). Gray boxes indicate the absence of particular satDNA in a corresponding genome. Predicted level of ploidy (2n, 3n, and 4n) [28, 37] for each species is indicated. For the abundance and repeat unit length of all 83 MelSat see Additional file 1: Table S1. The consensus sequences of all 83 MelSat are listed in Additional file 1: Table S5. MelSat01 corresponds to previously described INC satDNA [31], MelSat32 corresponds to MPA2 [11] and MelSat60, MelSat65, and MelSat76 represent previously published MARJA, MPA1, and AJL satDNAs respectively [12, 38, 39]

To identify the pool of satDNAs shared by multiple species and investigate their distribution during species evolution, we performed comparative satellitome analyses by simultaneous clustering of NGS data obtained for each species: *M. floridensis*, *M. incognita*, *M. arenaria*, and *M. javanica* (Figs. 1 and 2). Only the clusters denoted as high-confidence category of satDNAs were taken into consideration. SatDNA sequences (named MelSat(n)) from different species which grouped together based on their high sequence similarity are presented in Fig. 2. Although there is the possibility that portion of MelSat could remain unclassified and non-clustered in such low coverage analysis due to extremely low copy number of repeat units or/and high sequence variability, the comparative satellitome analyses point to the evolutionary trends of satDNAs in these related genomes.

**Fig. 3:**
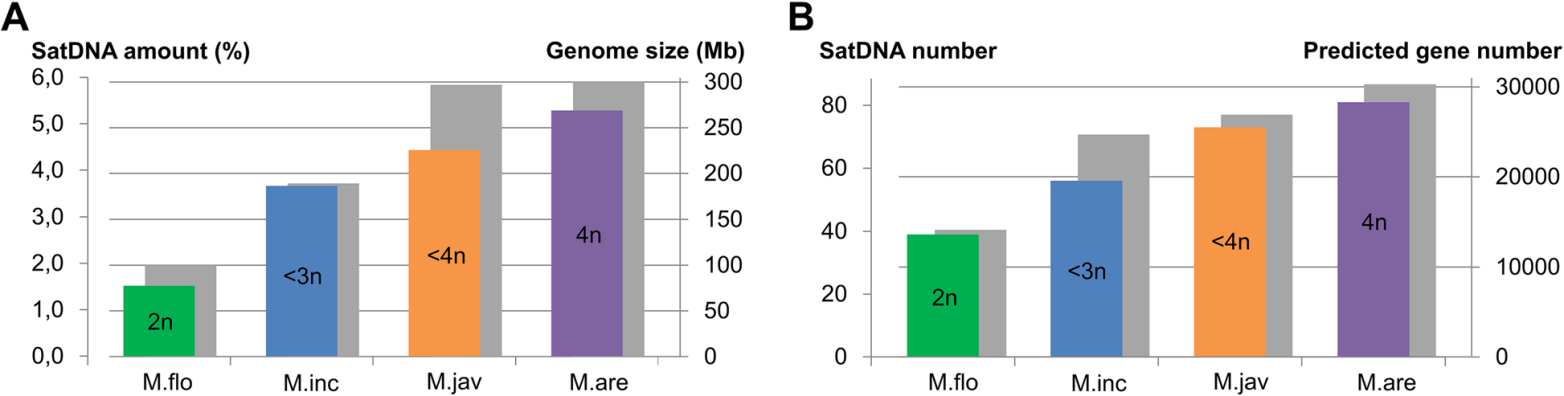
**A** Comparison between satDNA amount (colored bars) and genome size (gray bars). Genome size were estimated for *M. incognita* (M.inc), *M. arenaria* (M.are), and *M. javanica* (M.jav) by flow cytometry [28], while genome size for *M. floridensis* (M.flo) was appraised based on the assembled genome portion [37]. **B** Comparison between number of satDNAs (colored bars) and predicted number of genes [29] (gray bars) in four Meloidogyne species. Each bar is marked with predicted level of ploidy (2n, <3n, <4, and 4n)

Satellitome analyses disclosed a remarkable collection of different satDNAs in each genome, from 39 satDNAs (MelSat01-MelSat39) in *M. floridensis* to 81 satDNAs in *M. arenaria* (MelSat01-MelSat80+MelSat83). The abundance of satDNA was estimated from the number of reads which originate from a particular species. All MelSat DNAs cumulatively constitute from 1.6% of *M. floridensis* to 5.1% of *M. arenaria* genome, respectively (Additional file 1: Table S1). Sequences divergence among species-specific repeat unit consensus of individual MelSat DNA was up to 21.1% (e.g., MelSat33), with an average median value of 2.7% (Additional file 1: Table S1). Except for a few of them (i.e., MelSat 23, 24, 33, 47 and 61), comparative analysis of consensus within any particular MelSat DNA revealed their high sequence conservation. Comparative study based on distribution profile of MelSat DNAs in analyzed *Meloidogyne* species classified MelSat into 4 groups: those common to all species, those shared by three or two species and the last group that includes species-specific MelSat DNAs (Fig. 2). Furthermore, *M. floridensis*, which has been considered as diploid with hybrid origin [37] from two species, has 39 satDNAs in its satellitome. Besides this basic set shared by all genomes, an additional subset of 20 satDNAs was detected in *M. incognita*/*M. arenaria*/*M. javanica*, and a new subset of 21 satDNAs is characteristic for *M. arenaria*/*M. javanica* exclusively. Comparison of total genome abundance of MelSat DNAs and estimated genome size in analyzed *Meloidogyne* species revealed that the increase in satDNAs abundance is proportional to the increase in genome size (Fig. 3A). The similar proportionality was also found between the number of MelSat DNAs and estimated gene number (Fig. 3B). Number and abundance of MelSat satDNAs are also in accordance with proposed level of ploidy 2n, <3n, <4n, and 4n for *M. floridensis*, *M. incognita*, *M. javanica*, and *M. arenaria*, respectively.

Mapping of MelSat DNA specific for *M. incognita* on the reference genome assembly and on unassembled scaffolds of *M. incognita* revealed that MelSat DNA arrays are mostly located at the ends of scaffolds (data not shown) or are completely absent from the reference genome (Additional file 1: Table S2). On the other hand, the majority of MelSat arrays mapped on unassembled reads cover the entire reads length. In addition, we mapped MelSat on the recently published *M. arenaria* genome obtained by long-read PacBio sequencing [40], and also frequently found MelSat DNA arrays on the scaffolds ends (data not shown).

### Transcription analysis of satellitomes

To address genome-wide satDNA expression among species and throughout development we compare satDNA transcription patterns, using RNAseq data from *M. floridensis*, *M. incognita*, *M. arenaria* and *M. javanica* and different *M. incognita* life stages. To analyze whether there is any bias in quantification of MelSat transcripts due to experimental approaches, we first performed the analyses of MelSat transcriptome from two RNA-seq data sets obtained from different libraries of the same life stage in *M. incognita* (J2 stage) using the two different mappers, Bowtie2 and BBMap. The analyses revealed the high reproducibility of data of MelSat transcripts in different RNA-seq data and based on different mapping tools (Additional file 1: Fig. S3).

**Fig. 4:**
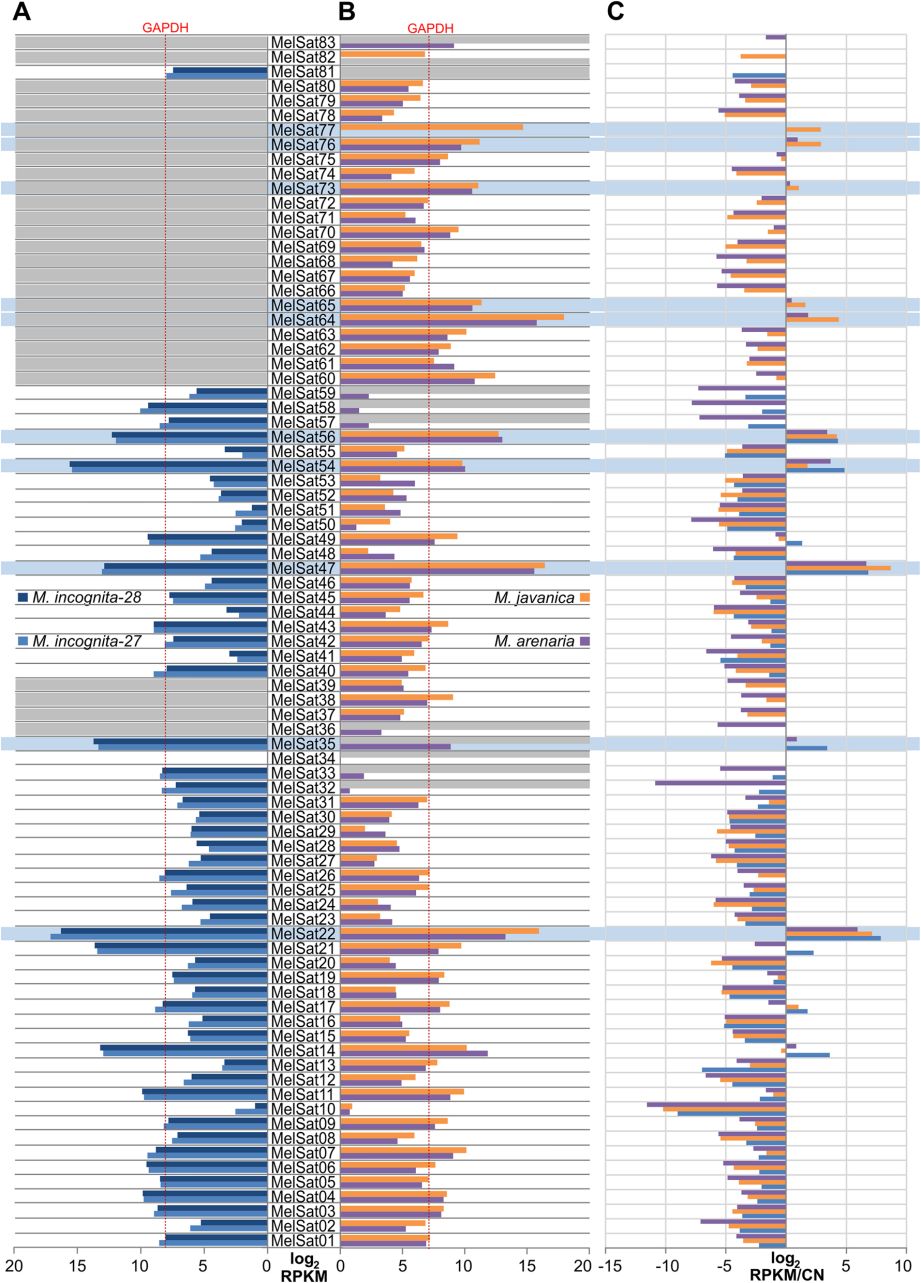
**A** Comparative analyses of MelSat DNAs expression in *M. incognita* (isolates 28 and 27), **B ***M. javanica* and *M. arenaria* using whole transcriptome data from [28]. We mapped the MelSat consensus sequences on the available RNA-seq data using Bowtie2 [41]. The quantification step included read counts and scaled read counts, and scaling method applied was log2RPKM (Reads Per Kilobase Million) (Additional file 1: Table S3). Expression profile was shown as logarithmic transformation of RPKM. Gray boxes show the absence of particular satRNA in the transcriptome. Red lines indicate the level of GAPDH gene expression. **C** The proportion of MalSat transcripts and genome abundance for each MelSat was calculated as a ratio of MalSat transcripts in respect to the total transcripts and copy number of MelSat in respect to genome sizes and length of satDNA repeat unit for each MelSat. Light blue boxes indicate MelSat DNAs with positive ratio in all species where MelSat were found. The expression analyses were also performed for 4 *M. incognita* housekeeping genes (Additional file 1: Table S4)

Transcription pattern of the two different isolates of *M. incognita* also show high reproducibility (Fig. 4A). In addition, we compared MelSat transcriptomes of *M. incognita*, *M. javanica* and *M. arenaria* mapping the transcriptomic libraries from RNA-seq data against the collection of MelSat consensus sequences (Fig. 4B). Expression analyses showed that the transcription pattern of the most MelSat RNAs shared by closely related *M. arenaria* and *M. javanica* are very similar (Fig. 4B). Moreover, MelSat RNAs which lack in *M. javanica* genome in comparison with *M. arenaria* show relatively low transcription level in *M. arenaria* (e.g. MelSat 32, 33, 34, 57, 58, 59). The only exception is MelSat 35 which is transcribed in *M. arenaria*, although it is absent in the *M. javanica* genome. A comparison of transcription profile of MelSat RNA common for *M. javanica/M. arenaria* group and *M. incognita*, shows overlapping expression pattern of MelSat shared by all three species (Fig. 4). To quantify observed similarity in MelSat transcription pattern among analyzed isolates/species we calculated correlation coefficients between MelSat transcriptomes of *M. incognita* isolates as well as transcriptomes between different species (Table 1). Correlation coefficient of satDNA transcriptomes between *M. incognita* isolates was approximately 0.99, which indicates a very high correlation within species. The analyses of correlation coefficients between species were also rather high, ranging from 0.71-0.83 indicating strong positive relationship. Since transcription level of the individual MelSat, especially those with low level of transcription, could be the result of passive transcription influenced by nearby genes whose expression levels might be conserved across species, we further investigated the proportion of MalSat transcripts and genome abundance for each MelSat in order to detect MelSat candidates which could be actively transcribed. The results are presented as ratio of MalSat transcripts in respect to the total transcripts and copy number of MelSat in respect to genome sizes and length of satDNA repeat unit for each MelSat (Fig. 4C). Although the results showed that the majority of MelSat have negative values and probably represent MelSat which are passively transcribed, 14 MelSat showed positive ratio and could be candidates for active transcription. Among them nine MelSat (22, 35, 47, 54, 56, 64, 65, 73, and 76) showed positive ratio in all species where they are present in the genome. The expression analyses of 4 house-keeping genes (Additional file 1: Table S4) showed expression level similar to highly transcribed MelSat.

**Table 1:** Evaluation of intraspecific (*M. incognita-27 and M. incognita-28*) and interspecific (*M. incognita-27/28*, *M. arenaria*, and *M. javanica*) transcription pattern correlation using Pearson correlation coefficient

*M. incognita-27* and *M. incognita-28*	*M. incognita-27* and *M. javanica*	*M. incognita-27* and *M. arenaria*	*M. arenaria* and *M. javanica*
0.986	0.816	0.712	0.809
	*M. incognita-28* and *M. javanica*	*M. incognita-28* and *M. arenaria*	
	0.834	0.743	

**Fig. 5:**
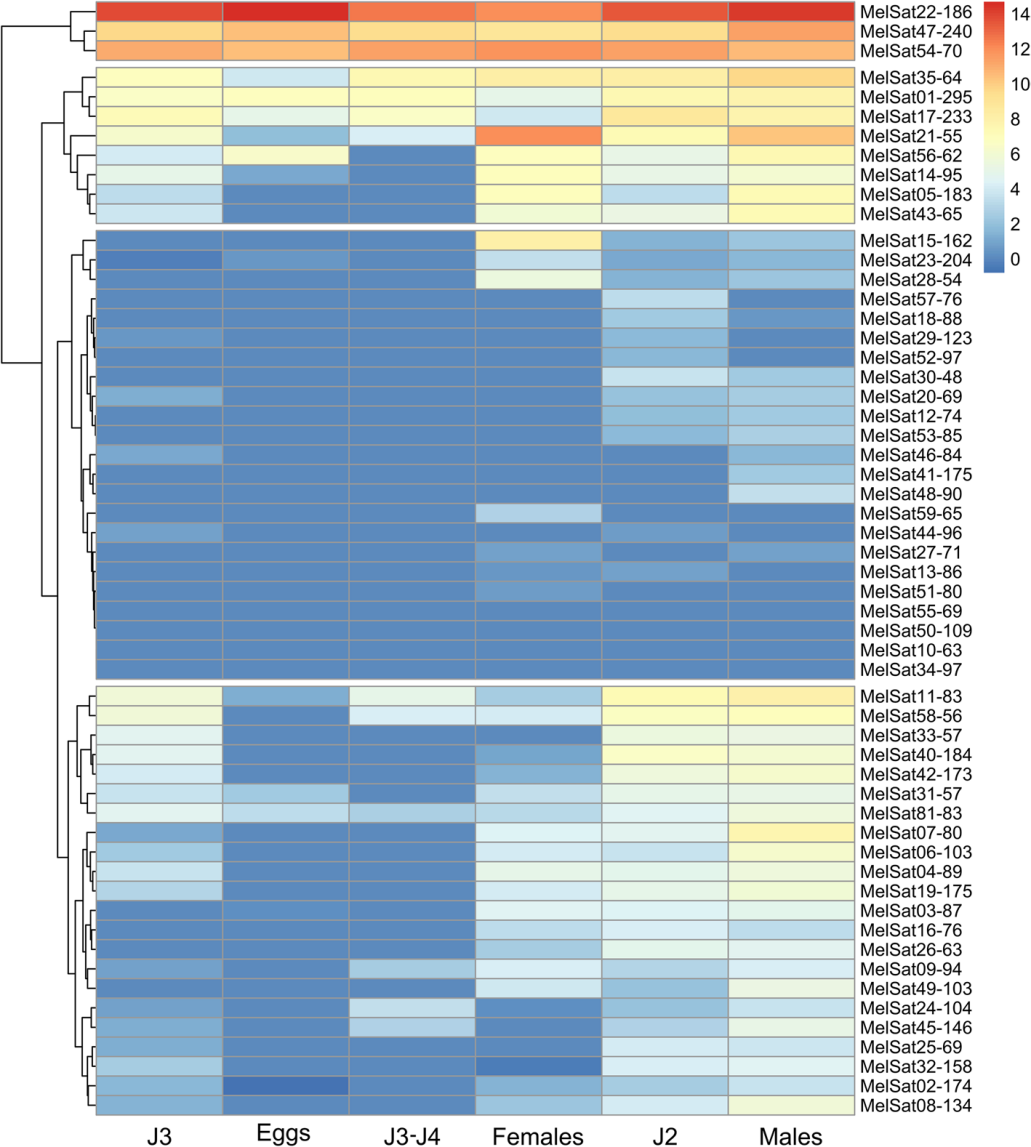
Hierarchical cluster analyses of MelSat satDNAs expression in different life stages (eggs, juveniles J2, J3, J3-J4, females and males) of *M. incognita* based on log ratio RPKM (Reads Per Kilobase Million) data. The quantification step included read counts, scaled read counts with applied log_2_RPKM scaling method. The tree branches indicate the four main clusters and the legend indicates satDNA family expression increase with colors going from blue to red

To gain insight into the developmental dynamics of satDNA expression we explore the pattern/level of MelSat transcription in different life stages, i.e., eggs, juveniles’ stages (J2, J3, J3-J4), male and female using RNA-seq data of *M. incognita*. The cluster analyses of stage-specific MelSat transcriptome analyses showed four clusters of MelSat (Fig. 5). Cluster I contains three MelSat with relatively high transcription in all developmental stages, while cluster II consists of 8 MelSat with moderate level transcription and absence of transcription in some developmental stages. The two largest clusters, III and IV, comprise MelSat which are extremely low transcribed or exhibit moderate/low level of transcripts in some stages. It is important to note that all five MelSat (22, 47, 54, 35, 56), found to be candidate for active transcription in *M. incogita* as well as in *M. arenaria* and *M. javanica* (Fig. 4C), are transcribed in all or almost all developmental stages (Fig. 5).

### MelSat DNAs sequences’ features

**Fig. 6:**
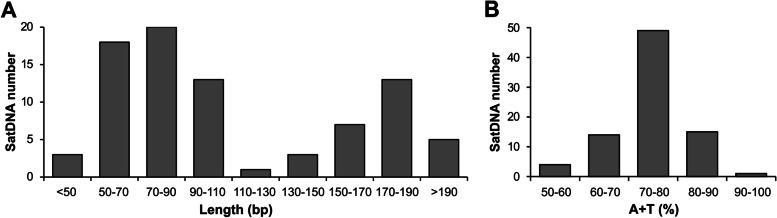
Repeat unit length distribution **A** and A+T content **B** of satDNAs. Analyses were performed on all 83 MelSat consensus repeat unit sequences from satellitomes of the analyzed *Meloidogyne* species. These and other main characteristics of all satDNAs are listed in Additional file 1: Table S1

The consensus sequences of MelSat DNAs are listed in Table S5. Among them, MelSat01 corresponds to the previously described INC satDNA [31], while MelSat32 corresponds to MPA2 [14]. MelSat60, MelSat65 and MelSat76 represent the previously published MARJA, MPA1, and AJL satDNAs, respectively [12, 38, 39], and were classified as MEL172 satDNA family [12]. The analyses of all 83 MelSat sequences disclosed variation of the repeat unit length from 30 bp to 300 bp (Additional file 1: Table S1). However, most MelSat repeat units (47 of 83) have a repeat unit from 50–90 bp and 170–190 bp (Fig. 6A) and high A+T content between 70% and 80% (Fig. 6B).

**Fig. 7:**
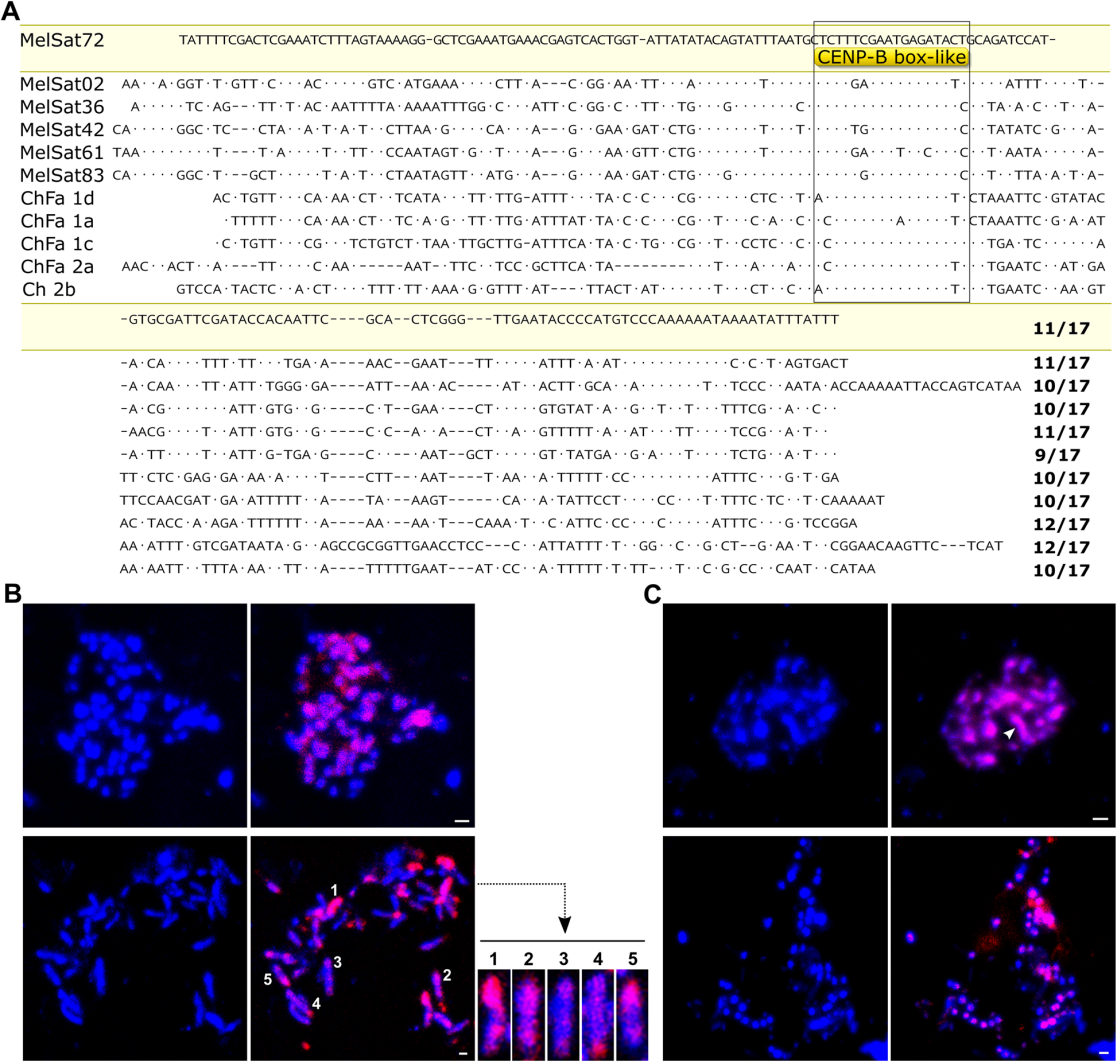
**A** Alignment of repeat unit consensus sequences of MelSat DNAs containing CENP-B box-like motif and ChFa (1d-2b) satDNAs from *M. chitwoodi* and *M. fallax* described previously [10]. CENP-B box-like motif is indicated within the boxed area. Nucleotide identities between *Meloidogyne* CENP-B box-like motif (reverse complement) and human CENP-B-box (YTTCGTTGGAARCGGGA; [13]) were provided for each satDNAs as a quotient on the right side of the alignment. **B** Fluorescence in situ hybridization of CENP-B box-like containing satDNAs (MelSat02 and MelSat42) (red signals) on *M. incognita* mitotic female chromosomes. Selected chromosomes were indicated with numbers. **C** Fluorescence in situ hybridization of CENP-B box-like containing satDNAs (MelSat02, MelSat36, MelSat42, MelSat61, MelSat72 and MelSat83) (red signals) on *M. arenaria* mitotic female chromosomes. Chromosomes are counter-stained with DAPI (blue). Scale bar: 1 μm

Comparison between consensus sequences of the 83 identified MelSat DNAs showed that most of them are not related. However, 14 MelSat DNAs based on sequence similarity could be sorted out into 6 different groups (Additional file 1: Fig. S4A-F). Four groups (MelSat11/70, MelSat 46/53, MelSat50/52 and MelSat74/79) include only two MelSat variants (Additional file 1: Fig. S4A-D). Three MelSat (60, 65, 76) (Additional file 1: Fig. S4E), belonging to the previously described MEL172 satDNA family [15], are characterized by repeat units with three different level of variability (Additional file 1: Fig. S4; E). A new group (comprising MelSat 42, 61, 83) with the CENP-B box like sequence previously found in distant species *M. chitwoodi* and M. *fallax* [10] was also detected (Additional file 1: Fig. S4; F). To test whether any additional MelSat containing CENP-B box-like motif exists in MIG species, we searched for it using BLAST against the 83 MelSat consensus sequences (Additional file 1: Table S5). Besides MelSat 42, 61 and 83, three new, more divergent MelSat DNAs (02, 36 and 72) with CENP-B box-like motif were also annotated. Among these six CENP-B box like-containing MelSat DNAs, one of them (MelSat 02) is shared by all four species, one (MelSat 42) is shared by three species, and one (MelSat83) is a species specific. The others (MelSat 36, 61, and 72) are common for two species (Figs. 2 and 7). The alignment of these six CENP-B box containing MelSat DNAs together with five satDNAs previously detected in distant *M. chitwoodi/M. fallax* shows conservation of a CENP-B box-like sequences (Fig. 7A). Namely, the average identity of CENP-B box-like sequences in analyzed satDNAs is 15 out of 17 nucleotides, which corresponds to 88% sequence similarity. On the contrary, overall repeat unit sequence identity among CENP-B box-like containing repeat units ranges from 36 to 74% (Additional file 1: Fig. S5A) suggesting high conservation of this sequence domain even in the highly divergent satDNAs and across the *Meloidogyne* genus. Interestingly, comparison of CENP-B box-like sequences with the human CENP-B box also shows relatively high degree of similarity. Among eleven CENP-B box-like sequences, ten of them have 10–12 out of 17 nucleotides conserved (Fig. 7A). In order to reveal chromosome localization of CENP-B box-containing MelSat DNAs, we performed FISH analysis on *M. incognita* and *M. arenaria* chromosomes. Since CENP-B box-containing MelSat DNAs are quite divergent (52–74%; Additional file 1: Fig. S5A), a mixture of probes specific for *M. incognita* (MelSat 02, 42) and those specific for *M. arenaria* (MelSat 02, 36, 42, 61, 72, 83) was used in FISH analyses (Additional file 1: Fig. S6). Interestingly, FISH results show localization of CENP-B box-containing MelSat DNAs on all chromosomes in both species (Fig. 7B, C). Their chromosome-specific localization shows holocentric distribution with different intensity, from weak to strong signals along the chromosomes (Fig. 7B).

**Fig. 8:**
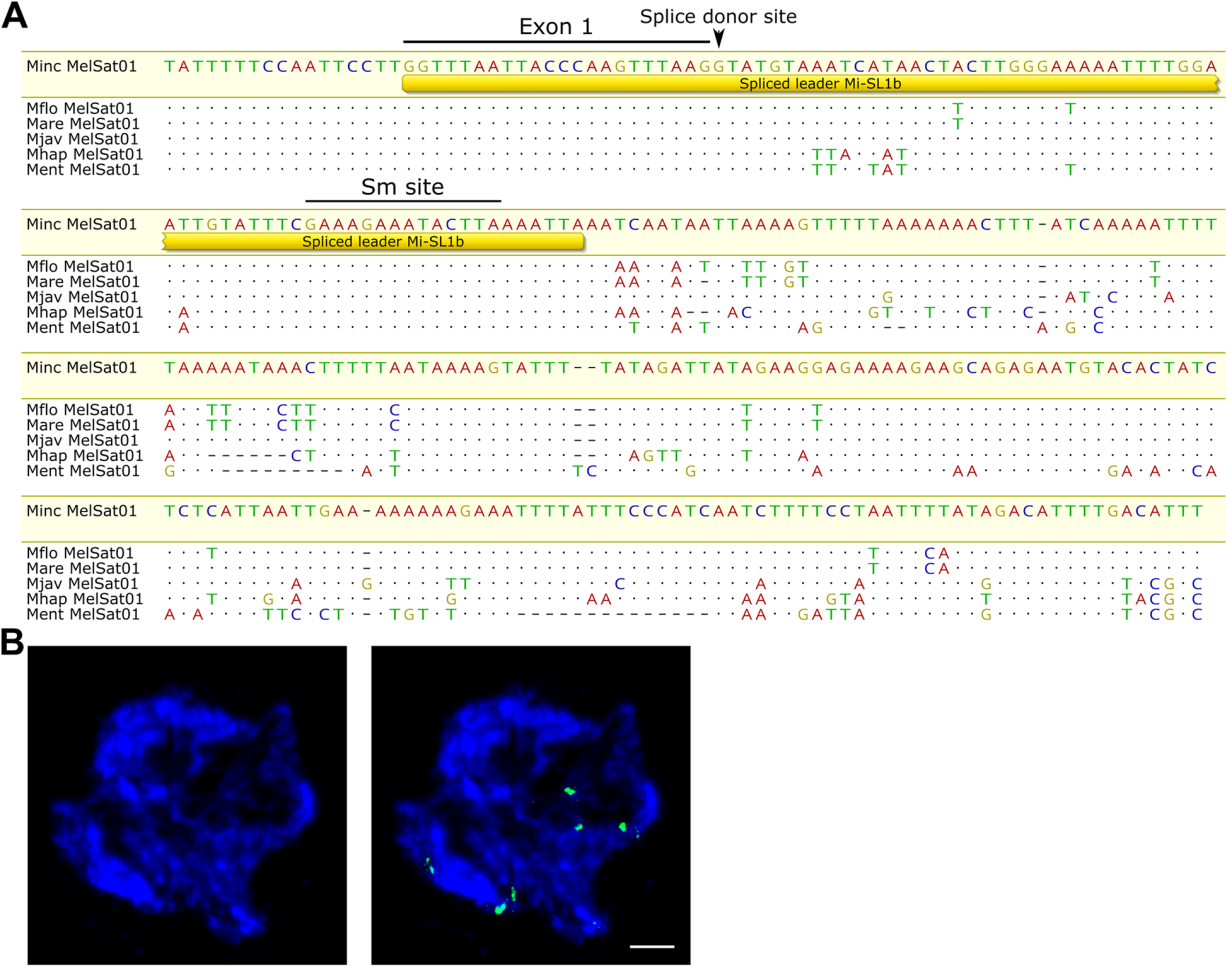
**A** Alignment of consensus sequences of MelSat01 from six *Meloidogyne* species (Minc-*M. incognita*, Mflo-*M. floridensis*, Mare-*M. arenaria*, Mjav-*M. javanica*, Mhap-*M. haplanaria*, and Ment-*M. enterolobii*). Yellow bar indicates position of spliced leader Mi-SL1b sequence with 22-bp SL exon region and Sm protein binding site. **B** Fluorescence in situ hybridization of MelSat01 (green signals) on *M. incognita* chromosomes. Chromosomes are counter stained with DAPI (blue). Scale bar: 1 μm

We further focused on MelSat01 (Fig. 2), previously described as INC satDNA [31], that has been found to correspond to Mi-SL1b splice leader sequence in *M. incognita* [42]. In order to investigate the trend of MelSat01 sequence change in related species, we compared MelSat01 consensus sequences, specific for particular species obtained from our satellitome analyses, with more distant *Meloidogyne* species, *M. enterolobii*, and *M. haplanaria* (Fig. 8A). The MelSat01 was extracted from *M. enterolobii* and *M. haplanaria* genome after graph-based clustering of publicly available Illumina sequence reads. The alignment of MelSat01 from all analyzed *Meloidogyne* species showed different sequence homology of MelSat01 repeat unit from 71 to 99% (Additional file 1: Fig. S5B) which is in accordance to species relationships. However, the pattern of variability along the repeat unit is characterized by low variability in the region which corresponds to splice leader Mi-SL1b sequence (about 90 bp in length), especially along the 22-bp SL exon region which includes splice donor site as well as along the sequence which is proposed as Sm protein binding site (Fig. 8A). In contrast, remaining part of the sequence shows higher variability particularly in the distant species *M. enterolobii* and *M. haplanaria*, with up to 30% sequence divergence (Additional file 1: Fig. S5B). Chromosome localization of MelSat01 in *M. incognita* using FISH analyses revealed prominent MelSat01 at six locations (Fig. 8B). In our cluster analyses through the *M. incognita* developmental stages MelSat01 belongs to the group of MelSat with moderate expression in almost all stages (Fig. 5, cluster II).

## Discussion

### Satellitomes illuminate evolutionary history of species

In the present work, we characterized and compared for the first time a whole complement of satDNAs (satellitome) in four *Meloidogyne* nematodes, *M. floridensis*, *M. incognita*, *M. arenaria*, and *M. javanica.* Our results revealed conservation of satDNA subsets shared by this group of related *Meloidogyne* species.

**Fig. 9:**
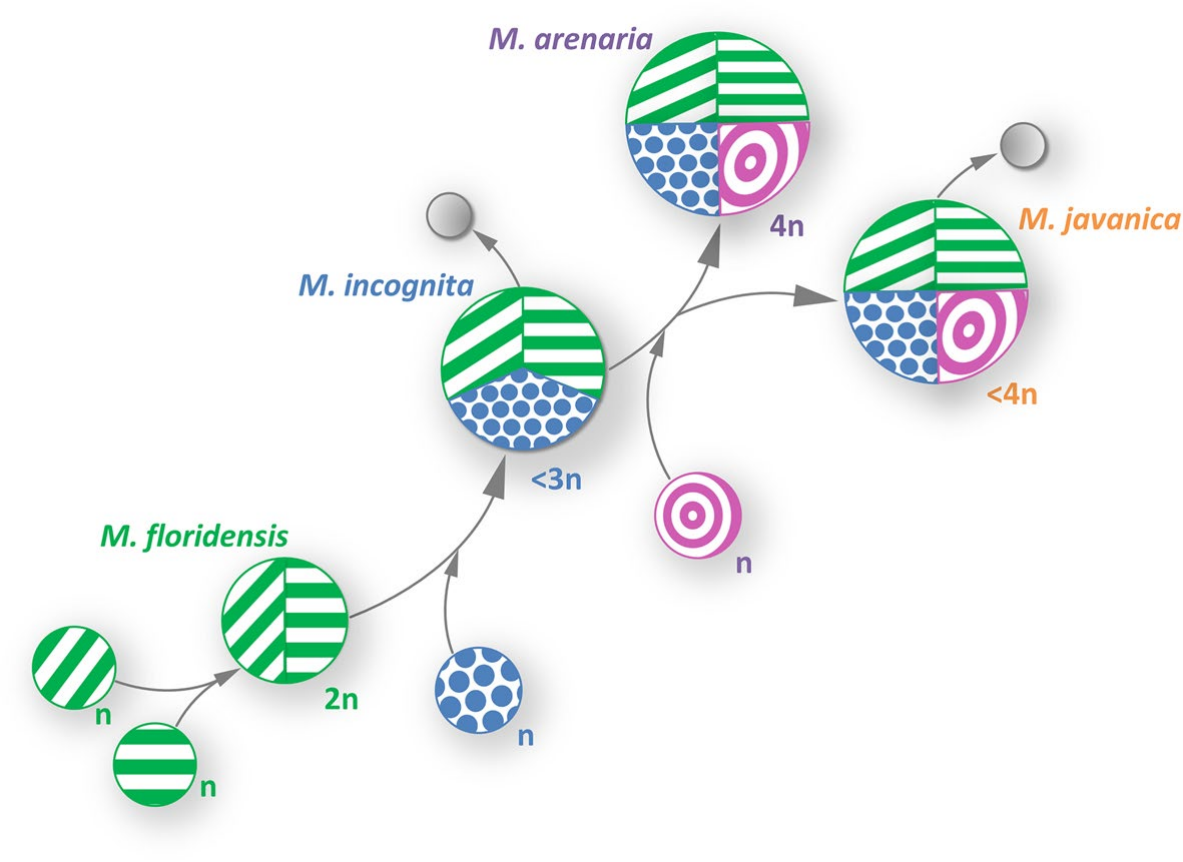
The most parsimonious evolutionary history of analyzed *Meloidogyne* species (*M.incognita*, *M. floridensis*, *M. arenaria and M. javanica*) according to our comparative analysis of satellitomes. Scheme represents successive hybridization events with the maternal recipient lineage and different closely related paternal donors (n). Grey circles indicate the loss of genomic parts which results in hypotriploid of *M. incognita* (<3n) and hypotetraploid genome of *M. javanica* (<4n)

A subset of 39 MelSat is common for all analyzed species and represents the basic satDNA set which is characteristic for *M. floridensis* genome (2n). An additional subset of 20 MelSat is shared by *M. incognita* (3n) and *M. arenaria*/*M. javanica* (4n) genomes, and a subset of 21 MelSat is present in *M arenaria/M. javanica* genomes only. The most important fact from the comparison of satellitomes in these species is that each round of proposed polyploidization is accompanied by addition of a new subset of MelSat DNAs in the satellitome of particular species which remains conserved in descendant species. Successive occurrence of the new set of satDNA in the process of polyploidization suggests multiple hybridization events as the main force in the formation of these species. We hypothesize that each hybridization event introduces a new set of MelSat emerged in closely related lineages during the species reproductive isolation (Fig. 9). Based on satellitome analyses, we further hypothesize the occurrence of at least two successive hybridization events with the maternal recipient lineage and different closely related paternal donors. Proportionality between number of MelSat DNAs and predicted gene number/level of polyploidization as well as positive linkage between MelSat DNA abundance and genome size also speak in favor of this hypothesis. Two recently published comparative studies of coding regions in these species offer different interpretations of ploidy. The comparative analysis of gene copies within the three mitotic *Meloidogyne* species suggests that their genomes have gone through several hybridization events, and consequently *M. incognita* is triploid, while *M. javanica* and *M. arenaria* are tetraploid [28]. On the other hand, the comparative study of coding regions in the three mitotic species (*M. incognita*, *M. arenaria*, and *M. javanica*) and meiotic *M. floridensis* based on divergence of gene copies proposed a diploid form reduced in gene number in the meiotic *M. floridensis* and a hypotriploid form in *M. incognita*, *M. javanica*, and *M. arenaria* [29].

We find that our studies of satellitomes, as non-coding genome parts that are subjected to evolutionary trends different than coding parts, successfully complement the studies made on the coding part. Inter-specific hybridization with at least two successive hybridization events between closely related lineages is the most parsimonious hypothesis that could resolve high turnover of overall satellitome profile between species (Fig. 9). Therefore, it could be concluded that new sets were not produced by divergence of duplicate genomes but are the product of hybridization of different but related genomes. As indicated previously, in mitotic and meiotic parthenogenetic *Meloidogyne* species, functional males occasionally occur in unfavorable environmental conditions [43]. Hypothetical hybridization events could occur in different meiotic or mitotic parthenogenetic species where species hybridization occurs by fertilization from related males bringing the new genome under unfavorable conditions. Our data also propound *M. floridensis* as an ancestral genome in the process of successive species hybridization events which is in accordance with the phylogenomic analysis of gene families in *M. floridensis*, *M. incognita*, and distant *M. hapla*, that highlighted *M. floridensis* as one of the putative parental species in the hybrid origin of *M. incognita* [37]. Furthermore, the absence of some satDNA from *M. incognita* and *M. javanica* satDNA subsets indicates the loss of genomic parts that followed the hybridization events (Fig. 9), which supports previously predicted hypotriploidy and hypotetraploidy in *M. incognita* and *M. javanica*, respectively [28]. Recent comparative analyses of satellitomes in two grasshoppers also found a high degree of shared satDNA families [7]. The authors provide a renewed view of satDNA evolution in the library which occurs through cycles of amplification and degradation [7].

### Transcription of MelSat

Since most species have an extremely abundant and, most often, species-specific satDNA in (peri)centromeric heterochromatin, a large number of studies on regulation, function and mechanisms of transcription have been made on that satDNA portion. It was shown that physiological transcription of (peri)centromeric satDNAs may be involved in different cell functions: for example, heterochromatin formation/silencing (reviewed in [21]), B chromosome drive [44], cell division [45], and regulation of genes [46]. However, due to species-specific and fast-evolving profile of satDNAs, genome-wide studies of satDNA-derived transcripts, and in particular comparative analyses of satDNA transcription in related genomes are very rare. Here, we present one of the most comprehensive analyses of satDNA transcription profiles on the genome-wide scale and among different species which was made possible by the existence of a large number of moderately amplified satDNAs and a common satDNA library in *Meloidogyne* species. We explored and compared transcription of 83 satDNAs in the three closely related *Meloidogyne* species and throughout life stages in *M. incognita.* Comparative analyses of satDNA transcriptome of the same life stage shared by the three species and correlation coefficients show relatively similar intra and interspecies transcription profile. Although our results indicate that most satDNAs in *Meloidogyne* species are silenced or passively transcribed, some of them show active transcription. Among actively transcribed satDNAs, the majority show active transcription in all the species in which genomes are found. This observation suggests that actively transcribed MelSat common for analyzed *Meloidogyne* species are in some way regulated rather than the result of a random event in closely related species. This hypothesis is supported by transcriptional pattern of these actively transcribed MelSat in different developmental stages of *M. incognita* which show high/moderate expression in almost all developmental stages. Since transcription alone does not directly imply a function, comparison of satDNAs transcription in different related species as well as during embryogenesis/development could be a way to address this issue. However, the possibility that satDNA transcription in some cases is a consequence of adjacent gene transcription cannot be completely ruled out. Recent studies disclosed that expression of satDNA-derived transcripts controls embryonal development in the mosquito via sequence-specific gene silencing [47]. SatDNA transcripts in piRNA (PIWI interacting RNAs)-dependent silencing regulate expression of a subset of genes in a sequence-dependent manner, suggesting function of satDNA in mosquito. The authors also point out that this satDNA locus was conserved for approximately 200 million years. Therefore, our analysis of orthologous satDNAs transcripts in related species could represent a strategy that can be also used in other species to provide knowledge of genome-wide regulation of satRNAs in general.

### SatDNAs with conserved sequence features

Contrary to satDNAs analyzed in the model nematode *C. elegans*, which do not show existence of any dominating repeat unit length [48], analyzed *Meloidogyne* species display clear predominance of ~80 bp or ~180 bp. This structural repeat unit feature is similar to the proposed length which can be linked to requirements for efficient DNA packing in chromatin [49]. Besides dominating repeat unit length, analyzed satDNAs also exhibit high AT content, as found in most satDNA in different animal and plant species [50]. Recently, it was shown that AT-rich satDNAs in *Drosophila* and mouse interact with AT-hook DNA-binding proteins, thus creating the architectural platform for the association of heterologous chromosomes in the single chromocenter [51].

Genome-wide survey and comparative sequence analyses of satDNAs offer a great opportunity to detect segments conserved in different satDNAs of particular species and among different species which could indicate possible functional competence. In analyzed *Meloidogyne* satellitomes, six quite divergent MelSat DNAs which contain CENP-B box-like motif previously described in satDNAs of the distant species *M. chitwoodi/M. fallax* were found [10]. The CENP-B box is a 17-bp long sequence conserved in alpha satDNAs of hominids. In human centromere, CENP-B protein binds the CENP-B box and plays an important role in the centromere assembly [52]. Interestingly, despite the high divergence among *Meloidogyne* CENP-B box-like containing satDNAs that rises up to 65%, when satDNAs from the distant species *M. fallax* and *M. chitwoodi* were included in the analysis, a 17 bp long CENP-B box-like motif turned out to be highly conserved. The *Meloidogyne* specific CENP-B box-like motif conserved among eleven divergent satDNAs shows relatively high homology with the human CENP-B box. DNA sequence motifs similar to the CENP-B box were also found in diverse mammalian species with similar level of identity to human CENP-B box as in *Meloidogyne* [53]. Based on these findings, we hypothesize that conservation of this CENP-B box-like sequence in highly divergent satDNAs of *Meloidogyne* species may be due to preservation of binding capacity for CENP-B-like protein. To date, CENP-B protein homologs were detected in many mammalian species, but not in other metazoans. However, transposase-derived proteins related to the CENP-B have been detected in diverse invertebrate and vertebrate species suggesting putative transposase activity of CENP-B-like proteins in these species [54, 55]. Moreover, the CENP-B box has been proposed to be involved in active mitotic recombination of alpha satDNA at the human centromere through the transposition-related mechanisms [56]. In that line, our previous results suggested a role of the CENP-B box-like sequence in the transposition-related mechanism of creation of complex satDNA arrays in *M. chitwoodi/M. fallax* [10]. Detection of conserved genes encoding CENP-B-like proteins in *M. incognita* [10] also supports the proposed hypothesis. Our recent study of centromere in MIG species disclosed CenH3 (centromeric histone H3)-associated centromeric DNA in a form of short arrays of tandem repeats (TRs), composed of five divergent families with conserved 19 bp box. The association of conserved 19 bp box with CenH3 histone suggests a role of conserved sequence motif of TRs in protein binding capacity [57]. The observed pattern of CENP-B-box-containing satDNAs distribution on the chromosomes in *M. incognita* was very similar to the distribution of *M. incognita* centromere characterized by highly abundant domains in different chromosomal regions [57] suggesting a putative role of CENP-B-box-containing satDNAs in organization of holocentric chromosomes. However, in order to explore mutual organization of centromeric TR and CENP-B-box-containing satDNAs in the context of long range organization of holocentromere and its surrounding areas, it will be essential to use long-read sequencing technologies.

In terms of other satDNAs with possible functional potential, MelSat01 found in four closely related and two distantly related *Meloidogyne* species represent Mi-SL1b, one of the two spliced leader (SL) gene variants previously described in *M. incognita*. SL genes are important in the trans-splicing process which stabilizes mRNA providing a 5′-cap structure, refines the 5′ untranslated region of pre-mRNA and enhances translation [58]. This process has been evidenced in a variety of eukaryotes, including nematodes. Two identified SL gene variants in *M. incognita*, Mi-SL1a and Mi-SL1b, although divergent in the sequences, possess two almost completely conserved regions, i.e., a 25-nt SL exon with splice donor site and a Sm binding consensus sequence required for the spliceosome activity. In nematodes, many mature mRNAs have this SL exon, and trans-splicing process is responsible for separating the long polycistronic pre-mRNAs [59]. Screening of SL1 transcripts in *M. incognita* EST database showed Mi-SL1a as the most frequently observed variant [42]. In order to investigate functional potential of MelSat01, we compared the consensus of repeat unit from closely related and distant *Meloidogyne* species and observed different levels of nucleotide variability across the MelSat01 sequences. Low variability is detected in SL gene sequence with complete conservation in the SL exon and Sm binding site implying a possible selection imposed on these functional regions. The rest of the repeat unit exhibits higher nucleotide variability especially between distant species. In addition, transcriptome analyses show that although MelSat01 does not belong to the group of actively transcribed satDNAs, it still shows moderate transcription in almost all developmental stages. The link between SL1 gene and satDNA in *Meloidogyne* species is similar to the one in *C. elegans* where about half of the genes are trans-spliced by SL1 genes which are organized in tandem repeats associated with the 5S RNA [60].

It can be assumed that conserved motifs such as CENP-B box-like motif and centromeric 19 bp box [57] found in highly evolved satDNAs of *Meloidogyne* may carry a functional signal in the form of a protein-binding site. In addition, conservation of satDNA sequence can also reflect a sequence-specific function as it can be assumed for satDNA-containing SL1 gene found conserved among *Meloidogyne*. In this regard, a new study on two complex satDNAs in *Aedes* provides strong evidence that short satDNA motif, conserved 200 Mya among mosquitos species, is responsible for the piRNA mediated sequence–specific gene silencing [47].

## Conclusions

Our analysis of the non-coding part of the genome that is primarily governed by different evolutionary trends in comparison to the coding part proved to be successful in elucidating species evolution. We consider that this methodology may be especially useful in groups of closely related species where standard phylogenetic markers do not contain a phylogenetic signal due to low sequence differentiation and/or in complex genomes subjected to a polyploidization process. Another feature that raised the question of the role of satDNA transcripts in cell physiological functioning are comparative studies of satDNA transcription among selected *Meloidogyne* species and through development that support the fact that transcription of some satDNAs could be subject to coordinated cell control in related species. Furthermore, the extensive analysis of satDNA sequences within and between species has enabled, despite satDNA rapid evolution, the discovery of conserved sequence features that are under selective pressure and could represent satDNA sequences with functional potential. We propose that active and coordinated transcription of some satDNAs in related genomes and across the development as well as conserved segments found in some satDNAs subsets indicate functional competence of some satDNAs in analyzed *Meloidogyne* species. Finally, due to genome abundance and repeat unit similarity, satDNAs are the most difficult part of a genome to sequence and assemble. Even the application of long-read sequencing technologies, which offered a substantial improvement for example in *M. arenaria* genome assembly, failed to assemble chromosome-length scaffolds due to gaps of satDNAs. Therefore, our data could shed some light on the current gaps occurring in *Meloidogyne* reference genomes.

## Methods

### DNA and RNA sequences sources

High-coverage Illumina short-read DNA data of *M. floridensis*, *M. incognita*, *M. arenaria*, and *M. javanica* were available from published sources [29], stating that DNA samples were extracted from J2 larvae, egg masses. Detailed sequencing data including geographic origin, name of isolate, as well as number and length of reads and genome coverage is listed in Additional file 1: Table S1 in [29]. For satellitome analyses, the raw whole genome sequences of four *Meloidogyne* species: *M. incognita* (isolates A14-SRR4242456, W1-SRR4242461 and L19-SRR4242479), *M. arenaria* (isolates HarA-SRR4242477, L32-SRR4242480 and L28-SRR4242481), *M. javanica* (isolates VW4-SRR4242459, L17-SRR4242471 and L15-SRR4242478), and *M. floridensis* (isolate SJF1-SRR4242475) were download from NCBI BioProject PRJNA340324 (https://github.com/HullUni-bioinformatics/MIG-Phylogenomics#mig-phylogenomics; [29]). Isolates were taken from diverse geographic locations and/or different plant species. Therefore, the isolates can be considered as different populations. Assembled genomes of *M. floridensis*, *M. incognita*, *M. javanica*, and *M. arenaria* were download from https://parasite.wormbase.org/.

Transcriptome analyses of satDNAs were done on RNA-seq data from NCBI BioProjects PRJEB8846, PRJEB8843, and PRJEB8845 [28, 61]. Briefly, total RNAs extracted from different species and developmental stages were provided to construct cDNA libraries using the Ovation Universal RNAseq system (Nugen technologies). Kit specifications declare that amplification is initiated at the 3′ end as well as randomly throughout the whole transcriptome in the sample. In this system, oligo dT primers are mixed with random primers for the first strand synthesis of cDNA products and provide RNA-Seq data from mRNA and non-polyadenylated transcripts. 28S and 18S rRNA transcripts were depleted using specific primers. Remaining ribosomal RNA contamination is eliminated using the program tool SortMeRNA.

For comparative analyses of satDNA transcriptome between species, paired-end reads (2x101bp) sequenced on the Illumina HiSeq2000 platform were used for *M. incognita* (ERR790027 and ERR790028; from J2.1 stage), *M. javanica* (ERR790020; from eggs and J2), and *M. arenaria* (ERR790021; from eggs and J2). For comparative analyses of satDNA transcriptome in different developmental stages RNA-seq data from juveniles J2 (ERR790026), juveniles J3 (ERR790029), parasitic juveniles J3-J4 (ERR790024), females (ERR790025), males (ERR790023), and eggs (ERR790022) of *M. incognita* were used. The transcripts of *M. incognita* developmental stages were sequenced on Illumina Genome Analyzer IIx, and generated single-end reads are for the downstream analysis filtered to 76 nt length. Statistics of sequencing technologies is provided in [28]. All transcriptomes were downloaded from https://www.ncbi.nlm.nih.gov/sra.

### SatDNA mining using graph-based clustering

The workflow of satDNA mining is presented in Fig. 1. Illumina WGS reads were checked for their quality using FastQC [62] and preprocessed by quality filtering, interlacing and random subsampling. Graph-based clustering was done using RepeatExplorer pipeline [34]. To find the optimal genome coverage with maximal number of repetitive DNA clusters but no single copy genes we tested several coverages (0.125, 0.25 and 0.5X) for each species. That range of genome coverage has been proposed in many previous satellitome studies [35]. In all analysis calculation of the genome coverage was done based on previous estimations of genome size for each species [28, 29]. Genome coverage of 0.25x was selected as optimal for all further comparative analyses. In addition, to test the reproducibility of satDNA characterization and distribution, RepeatExplorer clustering of three different isolates which were available for three species were performed. Due to the fact that species used in analysis have genomes of different sizes, in the comparative satellitomes’ analyses, we ensured the same coverage for each species by selecting subsample of certain size. Among satDNAs assigned as high-confidence, obtained clusters were manually checked for their graph shape, density and tandem organization of underling contigs. For each satDNA, genome abundancy in comparative studies was calculated by number of reads that contribute to cluster size divided by number of reads analyzed in that isolate or species [63]. Resulting comparative hit counts for all high confident satDNA were used for making four species repeat distribution graph. Detection threshold was set to 10 hits for positive satDNA presence in each species that was verified by manual inspection on existence of certain satDNA in each individual species analysis. Satellite clusters were sorted based on their shared appearance in smaller to larger genomes. Names of satDNAs were then assigned as suggested [1] with numbers according to their decreasing abundance in comparative analysis and MelSat as abbreviation for *Meloidogyne* genus satDNAs.

### Sequence analysis

SatDNA consensus sequences from multi-species comparative analysis were used as query for finding each satDNA in contigs of separate species clustering outputs. Repeat units for each satDNA were aligned and analyzed for pairwise identity and A+T content using Geneious v.9.1.8 program package. All satDNA consensus sequences were compared among themselves to find possible homology and only ones with similarity above 65% are considered as satDNA variants. Search for unique motifs was done with the tool fuzznuc from EMBOSS package using sequence of highly conserved region similar to previously described CENP-B box in *Meloidogyne* [10]. In order to look more deeply into the spliced leader (SL) sequence and its known association with satellite DNA [42], we also searched for Mi-SL1a and Mi-SL1b sequences. Multiple alignment of satDNAs with the found CENP-B box or SL motifs were performed with ClustalW [64].

### Analysis of the whole transcriptome data

Illumina HiSeq reads for *M. incognita*, *M. arenaria*, and *M. javanica* were filtered to length of 101 bp. Illumina Genome Analyzer IIx RNA-seq data from different *M. incognita* stages was preprocessed by filtering longest fraction of 76 bp reads where 9861803 eggs, 6764591 J2, 5629547 J3, 1819768 J3-J4, 8064588 females, and 7586720 male reads were subsequently analyzed. We were able to perform comparative analyses of MelSat transcription profile between species as well as between different stages since the same sequencing and bioinformatic approached were used. It is important to note that reverse transcription was previously performed [61] with the Ovation pico WTA System that uses mix of 3′ end and random primers, thus RNA-seq data represents whole transcriptome. Single-end reads were mapped to consensus sequences of satDNAs from species specific satelitomes for each life stage separately using Bowtie2 [41] v.2.3.0 with parameters -a and --very-sensitive. Reads were also mapped to GAPDH mRNA sequence (NCBI accession number BE191706) as one of reference genes that showed consistent and high expression in *Meloidogyne* across different stages of life cycle. For satDNAs shorter than 50 bp, mapping was done on concatenated repeat units until reaching 100 bp length. Output hits were sorted in Geneious and normalization by satDNA repeat unit length and number of mapped reads in RNA-seq library was performed with RPKM (reads per kilobase of transcript per million mapped reads) method. RPKM was calculated as a number of satellite hits divided by a number of mapped reads per million reads and repeat unit length in kilobase. SatDNAs are in graph sorted by their ascending catalog numbers where expression levels are shown as logarithmic transformation of RPKM values. Satellites that showed either high expression (relative to GAPDH) or differential expression were singled out. Separate analysis of the expression of housekeeping genes was performed in *M. incognita* based on previously validated candidates [65]. Reproducibility of our analysis was tested by comparing satDNA expression on two RNA-seq data from *M. incognita* juvenile stage 2 using the tools Bowtie2 and BBMap v35.82 [66]. Interspecific expression is shown for data from *M. arenaria* and *M. javanica* on mixed stages of eggs and J2.

In order to detect MelSat which are actively transcribed, ratio between transcription (RPKM) and genome abundance was performed. Genome abundance is calculated as MelSat copy number obtained from RepeatExplorer2 in respect to genome sizes for each species and length of satDNA repeat unit. Logarithmic values of these ratios are shown on the graph in Fig. 4C. Next, obtained log_2_RPKM values of satDNA transcripts are used for performing hierarchical clustering that was visualized with heatmap divided into four main groups based on the results of clustering. Drawing of clustered heatmap was done in R with the pheatmap package and default parameters except for cutree_rows argument which was set to four. Pearson correlation coefficient was used to evaluate transcription correlation between different species on the same data.

### Cytological validation

Genomic DNA from *M. incognita* and *M. arenaria* was isolated from egg phase with DNeasy Blood and Tissue Kit (Qiagen) followed by a RNA removal step with RNase A (Roche) for 10 min at 37 °C. MelSat01 (spliced leader-containing satDNA) dimer cloned in pGEM-T Easy vector was amplified and labeled directly using previously published specific primers [33]. MelSats that contain CENP-B box satDNAs (MelSat 02, 36, 61, 72 and 83), except MelSat42, were amplified and labeled directly from genomic DNA with specific primers listed in Table S6. MelSat42 was cloned because PCR labeling from genomic DNA did not provide sufficient level of probe specificity. PCR cycle for all probes comprised of 3 min initial denaturation at 95 °C followed by 35 cycles of 20 s denaturation at 95 °C, 20 s annealing at 55 °C, 40 s extensions at 72 °C, and final extension at 72 °C for 5 min. PCR labeling of FISH probes for MelSat01 and CENP-B box containing MelSat DNAs was done with biotin-16-dUTP and Cy3 dNTP mix (Jena Bioscience), respectively. PCR products were cleaned with PCR purification kit (Qiagen) and finally eluted in mqH_2_O and visualized on 1% agarose gel (Additional file 1: Fig. S6). In order to obtain chromosome spreads for FISH analysis, gonads were isolated from females extracted from infected tomato roots soaked in M9 buffer (22 mM KH_2_PO_4_, 42 mM Na_2_HPO_4_, 85.5 mM NaCl, 1 mM MgSO_4_). Gonads were collected in PBS buffer and gently crushed with electric homogenizer. Further, suspension was additionally homogenized in Dounce homogenizer with pestle A on ice for several minutes. Suspension was passed through 100 μm and 40 μm cell strainer before loading into Cytospin funnels and spun at 1000g for 10 min. Slides were post-fixed in acetone: methanol 1:1 for 20 min at − 20 °C, dried and stored at − 80 °C until later used. Five females’ gonads were usually enough for preparation of one slide.

Fluorescence in situ hybridization procedure was slightly modified for short AT-rich probes and highly condensed *Meloidogyne* chromosomes. Pre-hybridization included 10 min treatment with 45% acetic acid, 30 min incubation with 100 μg/mL RNase A at 37 °C, 10 min incubation with 100 μg/mL pepsin at 37 °C, and 10 min post-fixation step with formaldehyde. Chromosomes are denatured at 72 °C for 5 min and incubated overnight at 35 °C with 15 μL of hybridization solution per slide, containing 100 ng of each probe in 60% formamide, 2× SSC, 8% dextran sulphate, and 20 mM sodium phosphate. Post-hybridization washes were done at 35 °C in 50% formamide, 2× SSC. Slides were finally counterstained with DAPI (4′,6-diamidino-2-phenylindole), mounted in Mowiol and observed with confocal microscope (Leica TCS SP5). Pictures were analyzed using Image J and processed with Adobe Photoshop CS2.

## Supplementary Information

**Additional file 1: Fig. S1.** Different genome coverages (0,125X, 0,25X and 0,5X) applied in graph-based clustering analysis by Repeat Explorer with number of total obtained clusters on isolates used for comparative analysis; *M. incognita* L19 (SRR4242479), *M. javanica* VW4 (SRR4242459), equal parts of three isolates (HarA, L32, L28) for *M. arenaria* (SRR4242477, SRR4242480, SRR4242481) and *M. floridensis* SJF1 (SRR4242475). **Fig. S2.** Comparative analysis of satellitomes in three different isolates of (A) *M. incognita* (isolates 56, 61, 79), (B) *M. javanica* (isolates 59, 71, 78), and (C) *M. arenaria* (isolates 77, 80, 81). Each column represents putative satDNA, while the areas of the colored rectangles are proportional to the abundance of individual satDNA in the genome ranging from 0-0.594% in *M. incognita*, 0-0.649% *in M. javanica* and 0-0.694% in *M arenaria*. **Fig. S3.** Comparison of MelSat transcripts of the two different transcriptome data sets from *M. incognita* using the Bowtie2 and BBMap mapper. **Fig. S4.** Alignments of six MelSat groups which show significant mutual repeat unit similarity (A-F). MelSat60, MelSat65 and MelSat76 (E) represent previously published MARJA, MPA1 and AJL satDNAs respectively [12, 38, 39]. MV1, LV1, MV2, LV2 and HV indicate domains of MEL 172 satDNA described previously [12]. CENP-B box-like sequence (F) previously found in distant *M. chitwoodi* and *M. fallax* [10]. **Fig. S5.** (A) Identity matrix of CENP-B box containing satDNAs (MelSat 72/02/36/42/61/83 from MIG species and from *M. chitwoodi* and *M. fallax* (ChFa) previous published in [10]) and (B) identity matrix of MelSat01 in different *Meloidogyne* species (Minc-*M. incognita*, Mflo-*M. floridensis*, Mare-*M. arenaria*, Mjav-*M. javanica*, Ment-*M. enterolobii* and Mhap-*M. haplanaria*). **Fig. S6.** Electrophoresis of FISH probes after PCR labeling and purification; (A) biotin-labeled probe for MelSat01 (spliced leader) amplified from a cloned dimer [33] (B) six MelSat probes (CENP-B box containing) amplified and labeled with Cy3 from *M. arenaria* genomic DNA (MelSat02, 36, 61, 72, 83) and from a cloned dimer of MelSat42. **Table S1.** Main characteristics of the 83 satDNAs found in the genomes of *Meloidogyne* species by RepeatExplorer based on consensus sequences of satDNA family found in each of the analyzed species. The slash symbol in the divergency column indicates that this satDNA family is only present in one species. **Table S2.** Statistics of satDNAs from *M. incognita* satellitome mapped on genome assembly [42] and unplaced reads. **Table S3.** Satellite DNA transcription data of *Meloidogyne* species. SatDNA transcription was obtained with Bowtie2 mapping and normalization using RPKM (reads per kilobase of transcript per million mapped reads) method. SatDNA are ordered based on their catalog number as shown in Fig. 2. Other details are described in Methods section Analysis of the whole transcriptome data. **Table S4.** Expression analyses of 4 house-keeping genes in *M. incognita* based on previously validated candidates [65]. For mapping of two juvenile (J2) transcriptome databases (ERR790027 and ERR790028), coding sequences (CDS) of reference genes downloaded from WormBase ParaSite (https://parasite.wormbase.org/index.html) were used as listed *M. incognita* loci for each gene. **Table S5.** Consensus sequences of 83 satDNAs found in the genomes of the *Meloidogyne* species by RepeatExplorer. **Table S6.** Primers pairs used for MelSat amplification and labeling.

## Data Availability

All data generated or analyzed during this study are included in this published article, its supplementary information files, and publicly available repositories. The datasets analyzed in the study are available in the NCBI database under BioProjects PRJNA340324, PRJEB8846, PRJEB8843, and PRJEB8845.

## Abbreviations

satDNA: Satellite DNA
TE: Transposable element
RKN: Root-knot nematode
MIG: *M. incognita* group
piRNA: PIWI interacting RNA
CenH3: Centromeric histone H3
TR: Tandem repeat
SL: Spliced leader
RPKM: Reads per kilobase million
