# Supplementary material for: Satellitome analyses in nematodes illuminate complex species history and show conserved features in satellite DNAs
Source: BMC Biol. 2022 Nov 18;20:259. doi: 10.1186/s12915-022-01460-7 (PMC9673304; doi:10.1186/s12915-022-01460-7)
Supplement: Supplementary file 1 — Additional file 1: Fig. S1. Different genome coverages (0,125X, 0,25X and 0,5X) applied in graph-based clustering analysis by Repeat Explorer with number of total obtained clusters on isolates used for comparative analysis; M. incognita L19 (SRR4242479), M. javanica VW4 (SRR4242459), equal parts of three isolates (HarA, L32, L28) for M. arenaria (SRR4242477, SRR4242480, SRR4242481) and M. floridensis SJF1 (SRR4242475). Fig. S2. Comparative analysis of satellitomes in three different isolates of (A) M. incognita (isolates 56, 61, 79), (B) M. javanica (isolates 59, 71, 78), and (C) M. arenaria (isolates 77, 80, 81). Each column represents putative satDNA, while the areas of the colored rectangles are proportional to the abundance of individual satDNA in the genome ranging from 0-0.594% in M. incognita, 0-0.649% in M. javanica and 0-0.694% in M arenaria. Fig. S3. Comparison of MelSat transcripts of the two different transcriptome data sets from M. incognita using the Bowtie2 and BBMap mapper. Fig. S4. Alignments of six MelSat groups which show significant mutual repeat unit similarity (A-F). MelSat60, MelSat65 and MelSat76 (E) represent previously published MARJA, MPA1 and AJL satDNAs respectively [12, 38, 39]. MV1, LV1, MV2, LV2 and HV indicate domains of MEL 172 satDNA described previously [12]. CENP-B box-like sequence (F) previously found in distant M. chitwoodi and M. fallax [10]. Fig. S5. (A) Identity matrix of CENP-B box containing satDNAs (MelSat 72/02/36/42/61/83 from MIG species and from M. chitwoodi and M. fallax (ChFa) previous published in [10]) and (B) identity matrix of MelSat01 in different Meloidogyne species (Minc-M. incognita, Mflo-M. floridensis, Mare-M. arenaria, Mjav-M. javanica, Ment-M. enterolobii and Mhap-M. haplanaria). Fig. S6. Electrophoresis of FISH probes after PCR labeling and purification; (A) biotin-labeled probe for MelSat01 (spliced leader) amplified from a cloned dimer [33] (B) six MelSat probes (CENP-B box containing) amplified and [file 12915_2022_1460_MOESM1_ESM.docx]

**Figure S1**. Different genome coverages (0,125X, 0,25X and 0,5X) applied in graph-based clustering analysis by Repeat Explorer with number of total obtained clusters on isolates used for comparative analysis; *M. incognita* L19 (SRR4242479), *M. javanica* VW4 (SRR4242459), equal parts of three isolates (HarA, L32, L28) for *M. arenaria* (SRR4242477, SRR4242480, SRR4242481) and *M. floridensis* SJF1 (SRR4242475).

**A**


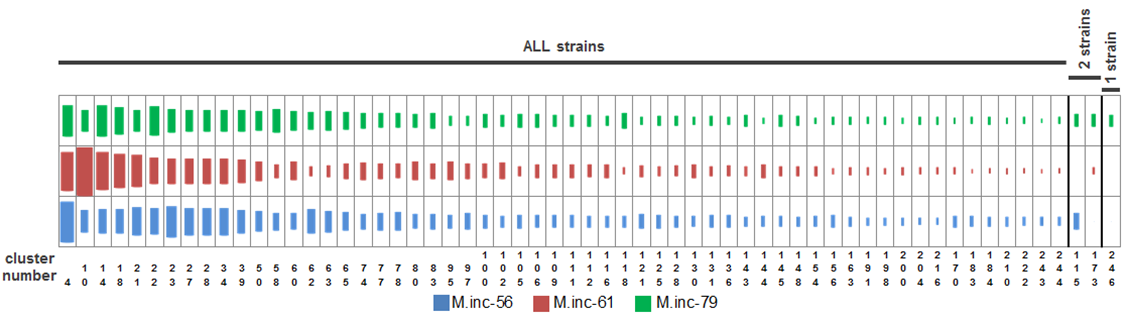


**B**


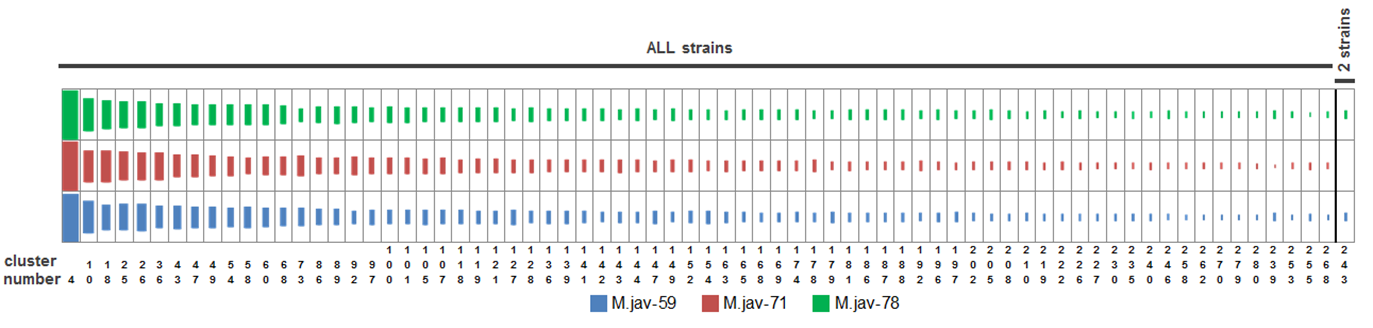


**C**


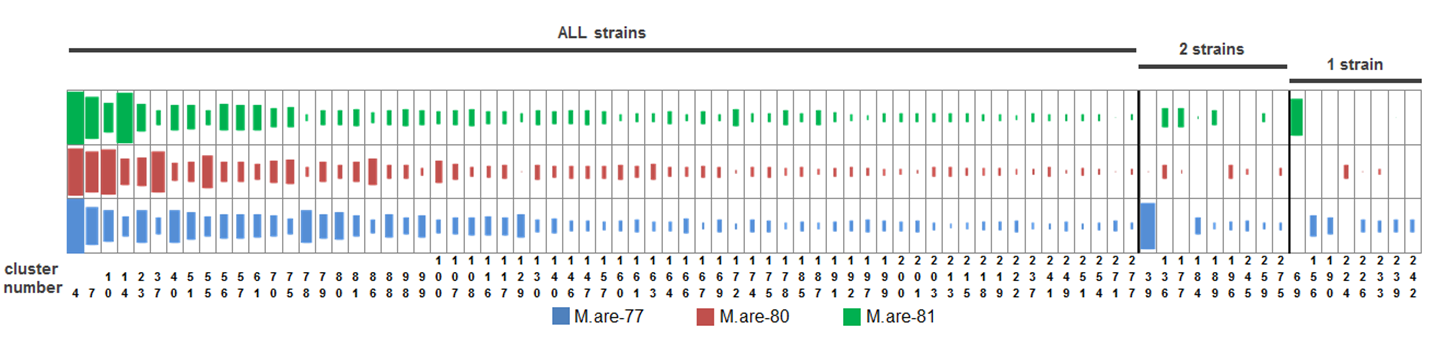


**Figure S2.** Comparative analysis of satellitomes in three different isolates of **(A)** *M. incognita* (isolates 56, 61, 79), **(B)** *M. javanica* (isolates 59, 71, 78), and **(C)** *M. arenaria* (isolates 77, 80, 81). Each column represents putative satDNA, while the areas of the colored rectangles are proportional to the abundance of individual satDNA in the genome ranging from 0-0.594% in *M. incognita*, 0-0.649% *in M. javanica* and 0-0.694% in *M arenaria*.

**
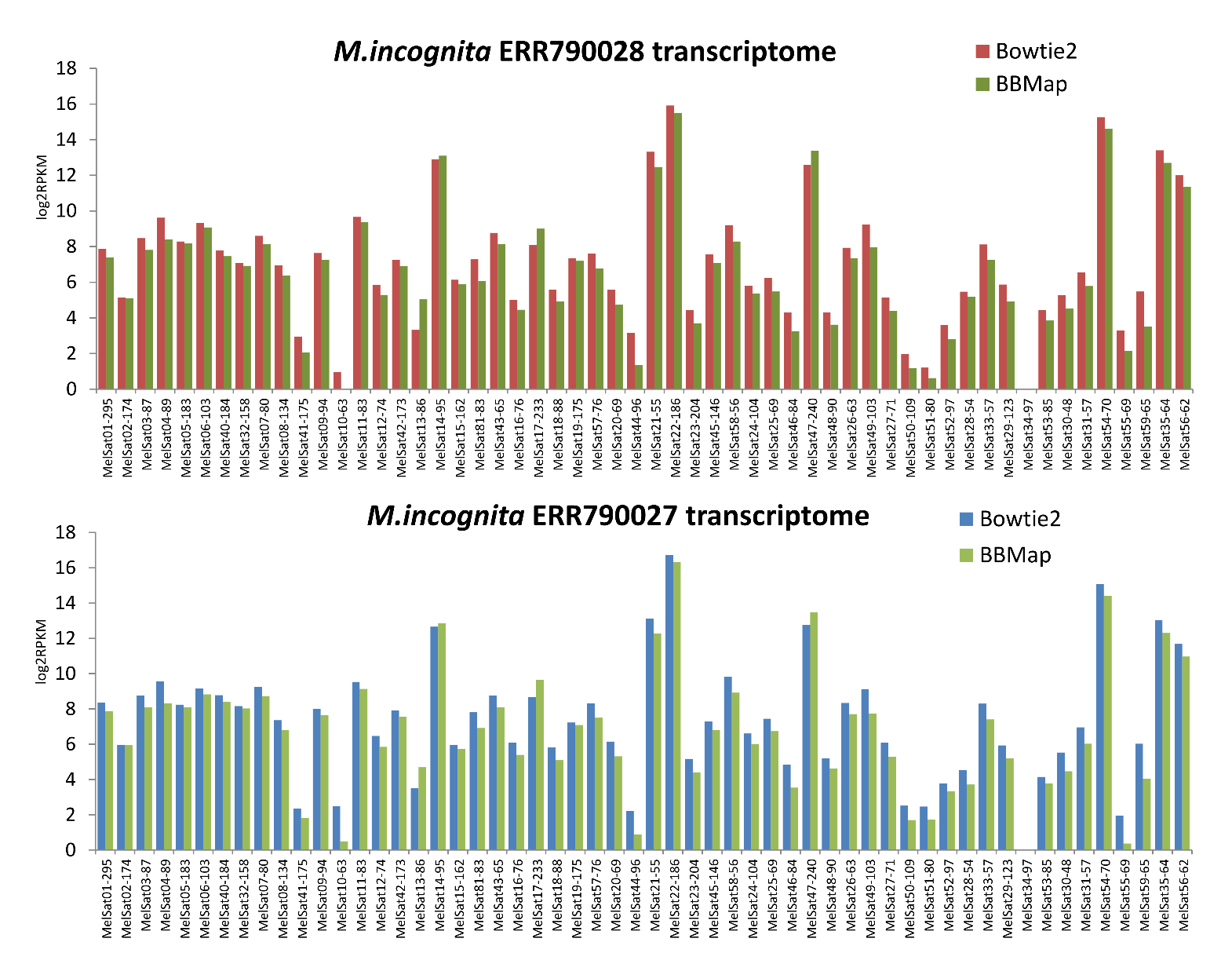
**

**Figure S3.** Comparison of MelSat transcripts of the two different transcriptome data sets from *M. incognita* using the Bowtie2 and BBMap mapper.

**A**

MelSat11/70 (71% sequence identity)


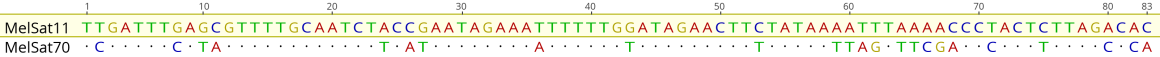


**B**

MelSat46/53 (81% sequence identity)


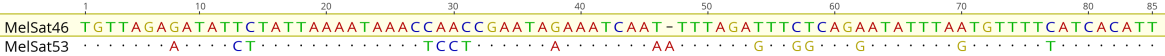


**C**

MelSat50/52 (68% sequence identity)


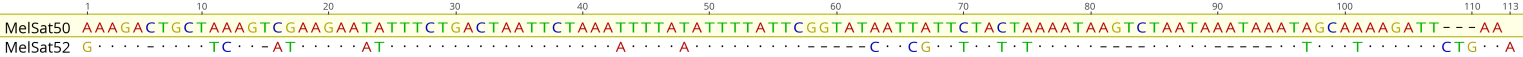


**D**

MelSat74/79 (63% sequence identity)


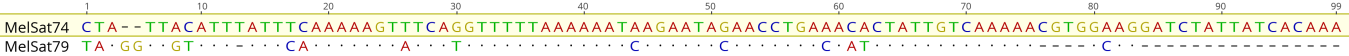


**E**

MEL 172 family (67% sequence identity)

MV2

HV

LV2

MV1

LV1


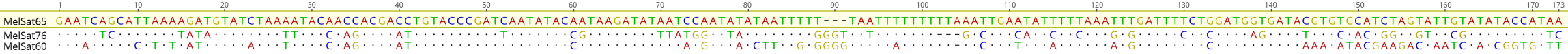


**F**

CENP-B box like

CENP-B box like containing MelSat variants (PI 75%)


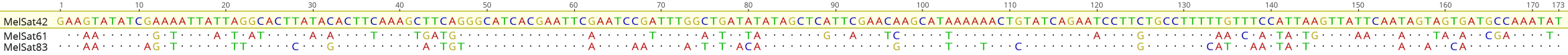


**Figure S4.** Alignments of six MelSat groups which show significant mutual repeat unit similarity (**A-F**). MelSat60, MelSat65 and MelSat76 (**E**) represent previously published MARJA, MPA1 and AJL satDNAs respectively [12, 38, 39]. MV1, LV1, MV2, LV2 and HV indicate domains of MEL 172 satDNA described previously [12]. CENP-B box-like sequence (**F**) previously found in distant *M. chitwoodi* and *M. fallax* [10].

**A**

**B**

**Figure S5. (A)** Identity matrix of CENP-B box containing satDNAs (MelSat 72/02/36/42/61/83 from MIG species and from *M. chitwoodi* and *M. fallax* (ChFa) previous published in [10]) and **(B)** identity matrix of MelSat01 in different *Meloidogyne* species (Minc-*M. incognita*, Mflo-*M. floridensis*, Mare-*M. arenaria*, Mjav-*M. javanica*, Ment-*M. enterolobii* and Mhap-*M. haplanaria*).

**
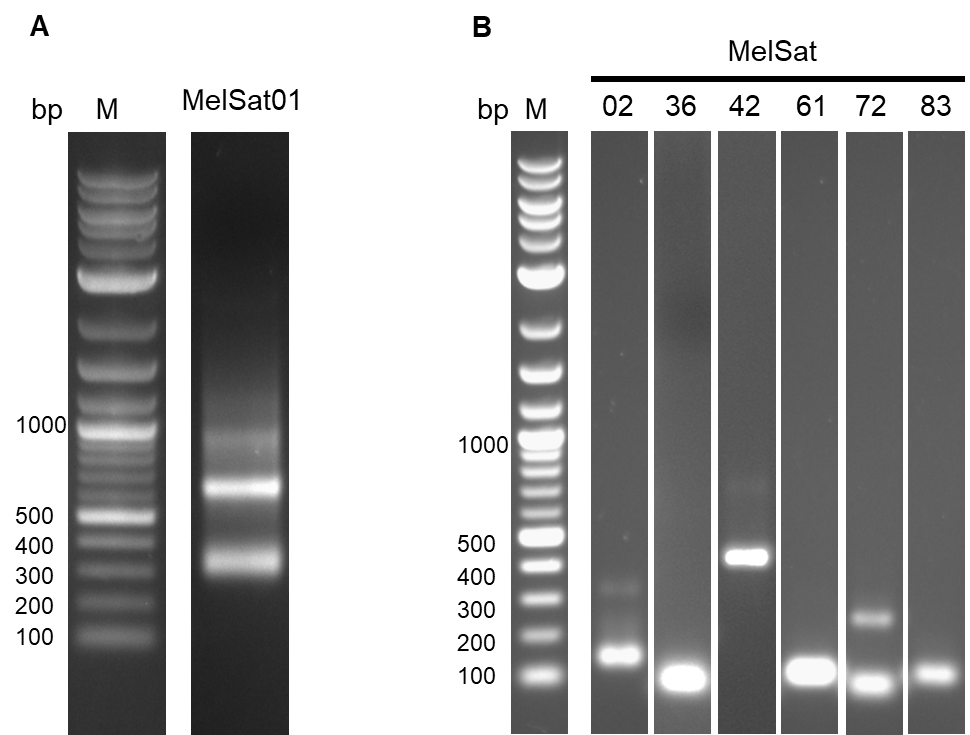
**

**Figure S6**. Electrophoresis of FISH probes after PCR labeling and purification; **(A)** biotin-labeled probe for MelSat01 (spliced leader) amplified from a cloned dimer [33] **(B)** six MelSat probes (CENP-B box containing) amplified and labeled with Cy3 from *M. arenaria* genomic DNA (MelSat02, 36, 61, 72, 83) and from a cloned dimer of MelSat42.


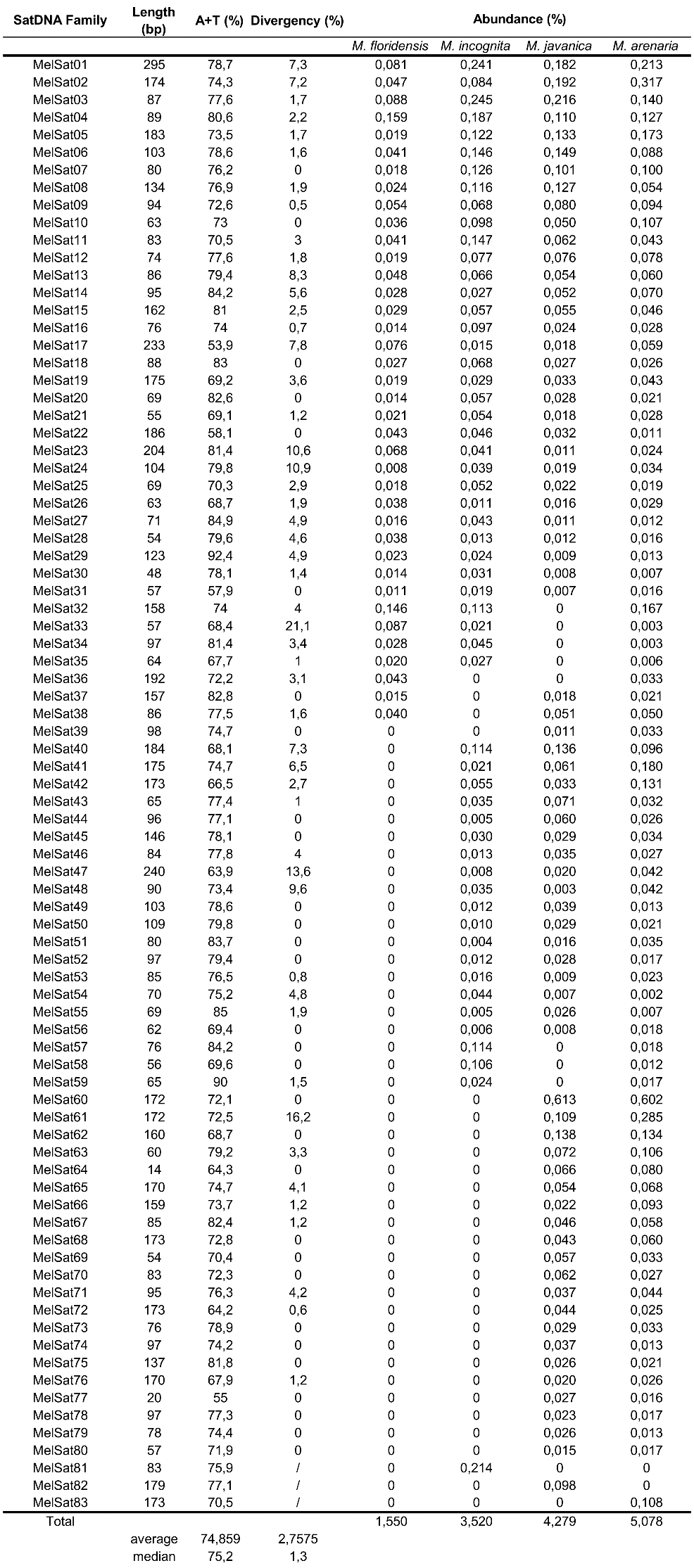
**Table S1.** Main characteristics of the 83 satDNAs found in the genomes of *Meloidogyne* species by RepeatExplorer based on consensus sequences of satDNA family found in each of the analyzed species. The slash symbol in the divergency column indicates that this satDNA family is only present in one species.

**Table S2**. Statistics of satDNAs from *M. incognita* satellitome mapped on genome assembly [42] and unplaced reads.

|  | **Meloidogyne_incognita_V3 genome assembly** | **MiV1_unplaced_reads** |
| --- | --- | --- |
| **number of scaffolds/reads** | 12091 | 267656 |
| **mean length of scaffolds/reads (bp)** | 15179 | 615 |
| **number of mapped scaffolds/reads** | 159 | 3310 |
| **number of mapped satDNAs/all satDNAs** | 45/56 | 56/56 |
| **total number of mapped monomers** | 1007 | 15157 |
| **proportion of satDNA (%)** | 0,06 | 1 |

**Table S3.** Satellite DNA transcription data of *Meloidogyne* species. SatDNA transcription was obtained with Bowtie2 mapping and normalization using RPKM (reads per kilobase of transcript per million mapped reads) method. SatDNA are ordered based on their catalog number as shown in Figure 2. Other details are described in Materials and methods section Analysis of the whole transcriptome data

|  |  | **Bowtie2 hits** | | | | **RPKM** | | | | **log2RPKM** | | | |
| --- | --- | --- | --- | --- | --- | --- | --- | --- | --- | --- | --- | --- | --- |
| **satDNA** | **length** | ***M. incognita-27*** | ***M. incognita-28*** | ***M.javanica*** | ***M. arenaria*** | ***M. incognita-27*** | ***M. incognita-28*** | ***M.javanica*** | ***M. arenaria*** | ***M. incognita-27*** | ***M. incognita-28*** | ***M.javanica*** | ***M. arenaria*** |
| **MelSat01-295** | 295 | 1091 | 1122 | 739 | 687 | 327.27 | 233.98 | 154.72 | 125.31 | 8.35 | 7.87 | 7.27 | 6.97 |
| **MelSat02-174** | 174 | 123 | 100 | 338 | 129 | 62.55 | 35.36 | 119.97 | 39.89 | 5.97 | 5.14 | 6.91 | 5.32 |
| **MelSat03-87** | 87 | 423 | 502 | 471 | 471 | 430.25 | 354.97 | 334.36 | 291.30 | 8.75 | 8.47 | 8.39 | 8.19 |
| **MelSat04-89** | 89 | 756 | 1135 | 577 | 550 | 751.68 | 784.53 | 400.41 | 332.52 | 9.55 | 9.62 | 8.65 | 8.38 |
| **MelSat05-183** | 183 | 628 | 927 | 422 | 336 | 303.68 | 311.63 | 142.42 | 98.79 | 8.25 | 8.28 | 7.15 | 6.63 |
| **MelSat06-103** | 103 | 668 | 1084 | 350 | 134 | 573.91 | 647.44 | 209.87 | 70.00 | 9.16 | 9.34 | 7.71 | 6.13 |
| **MelSat07-80** | 80 | 553 | 507 | 1551 | 849 | 611.70 | 389.87 | 1197.39 | 571.03 | 9.26 | 8.61 | 10.23 | 9.16 |
| **MelSat08-134** | 134 | 249 | 268 | 140 | 62 | 164.44 | 123.04 | 64.53 | 24.90 | 7.36 | 6.94 | 6.01 | 4.64 |
| **MelSat09-94** | 94 | 272 | 305 | 639 | 359 | 256.06 | 199.61 | 419.84 | 205.50 | 8.00 | 7.64 | 8.71 | 7.68 |
| **MelSat10-63** | 63 | 4 | 2 | 2 | 2 | 5.62 | 1.95 | 1.96 | 1.71 | 2.49 | 0.97 | 0.97 | 0.77 |
| **MelSat11-83** | 83 | 691 | 1093 | 1417 | 751 | 736.72 | 810.12 | 1054.40 | 486.86 | 9.52 | 9.66 | 10.04 | 8.93 |
| **MelSat12-74** | 74 | 74 | 70 | 82 | 43 | 88.49 | 58.19 | 68.44 | 31.27 | 6.47 | 5.86 | 6.10 | 4.97 |
| **MelSat13-86** | 86 | 11 | 14 | 326 | 195 | 11.32 | 10.01 | 234.12 | 122.01 | 3.50 | 3.32 | 7.87 | 6.93 |
| **MelSat14-95** | 95 | 6970 | 11772 | 1888 | 7042 | 6492.48 | 7623.09 | 1227.42 | 3988.56 | 12.66 | 12.90 | 10.26 | 11.96 |
| **MelSat15-162** | 162 | 114 | 186 | 127 | 120 | 62.27 | 70.63 | 48.42 | 39.86 | 5.96 | 6.14 | 5.60 | 5.32 |
| **MelSat16-76** | 76 | 58 | 40 | 36 | 46 | 67.53 | 32.38 | 29.26 | 32.57 | 6.08 | 5.02 | 4.87 | 5.03 |
| **MelSat17-233** | 233 | 1069 | 1031 | 1756 | 1191 | 406.00 | 272.21 | 465.46 | 275.04 | 8.67 | 8.09 | 8.86 | 8.10 |
| **MelSat18-88** | 88 | 56 | 69 | 32 | 38 | 56.31 | 48.24 | 22.46 | 23.24 | 5.82 | 5.59 | 4.49 | 4.54 |
| **MelSat19-175** | 175 | 296 | 462 | 982 | 827 | 149.68 | 162.41 | 346.57 | 254.28 | 7.23 | 7.34 | 8.44 | 7.99 |
| **MelSat20-69** | 69 | 55 | 54 | 18 | 29 | 70.54 | 48.14 | 16.11 | 22.61 | 6.14 | 5.59 | 4.01 | 4.50 |
| **MelSat21-55** | 55 | 5554 | 9171 | 804 | 257 | 8936.03 | 10257.90 | 902.83 | 251.43 | 13.13 | 13.32 | 9.82 | 7.97 |
| **MelSat22-186** | 186 | 226887 | 187250 | 213256 | 37247 | 107943.89 | 61931.78 | 70811.29 | 10775.13 | 16.72 | 15.92 | 16.11 | 13.40 |
| **MelSat23-204** | 204 | 83 | 72 | 31 | 70 | 36.00 | 21.71 | 9.39 | 18.46 | 5.17 | 4.44 | 3.23 | 4.21 |
| **MelSat24-104** | 104 | 115 | 94 | 14 | 33 | 97.85 | 55.60 | 8.31 | 17.07 | 6.61 | 5.80 | 3.06 | 4.09 |
| **MelSat25-69** | 69 | 136 | 85 | 161 | 91 | 174.42 | 75.78 | 144.11 | 70.96 | 7.45 | 6.24 | 7.17 | 6.15 |
| **MelSat26-63** | 63 | 231 | 250 | 155 | 98 | 324.47 | 244.12 | 151.95 | 83.70 | 8.34 | 7.93 | 7.25 | 6.39 |
| **MelSat27-71** | 71 | 54 | 41 | 9 | 9 | 67.30 | 35.52 | 7.83 | 6.82 | 6.07 | 5.15 | 2.97 | 2.77 |
| **MelSat28-54** | 54 | 14 | 39 | 21 | 28 | 22.94 | 44.43 | 24.02 | 27.90 | 4.52 | 5.47 | 4.59 | 4.80 |
| **MelSat29-123** | 123 | 85 | 117 | 8 | 29 | 61.15 | 58.52 | 4.02 | 12.69 | 5.93 | 5.87 | 2.01 | 3.67 |
| **MelSat30-48** | 48 | 25 | 30 | 14 | 14 | 46.09 | 38.45 | 18.01 | 15.69 | 5.53 | 5.26 | 4.17 | 3.97 |
| **MelSat31-57** | 57 | 80 | 87 | 122 | 86 | 124.20 | 93.90 | 132.19 | 81.18 | 6.96 | 6.55 | 7.05 | 6.34 |
| **MelSat32-158** | 158 | 509 | 346 |  | 5 | 285.08 | 134.72 |  | 1.70 | 8.16 | 7.07 |  | 0.77 |
| **MelSat33-57** | 57 | 203 | 258 |  | 4 | 315.15 | 278.45 |  | 3.78 | 8.30 | 8.12 |  | 1.92 |
| **MelSat34-97** | 97 | 0 | 0 |  | 0 | 0.00 | 0.00 |  | 0.00 | 0.00 | 0.00 |  | 0.00 |
| **MelSat35-64** | 64 | 6079 | 11286 |  | 588 | 8405.31 | 10848.38 |  | 494.36 | 13.04 | 13.41 |  | 8.95 |
| **MelSat36-192** | 192 |  |  |  | 36 |  |  |  | 10.09 |  |  |  | 3.33 |
| **MelSat37-157** | 157 |  |  | 91 | 85 |  |  | 35.80 | 29.13 |  |  | 5.16 | 4.86 |
| **MelSat38-86** | 86 |  |  | 786 | 212 |  |  | 564.47 | 132.64 |  |  | 9.14 | 7.05 |
| **MelSat39-98** | 98 |  |  | 50 | 64 |  |  | 31.51 | 35.14 |  |  | 4.98 | 5.14 |
| **MelSat40-184** | 184 | 913 | 657 | 360 | 156 | 439.09 | 219.66 | 120.84 | 45.62 | 8.78 | 7.78 | 6.92 | 5.51 |
| **MelSat41-175** | 175 | 10 | 22 | 180 | 103 | 5.06 | 7.73 | 63.53 | 31.67 | 2.34 | 2.95 | 5.99 | 4.99 |
| **MelSat42-173** | 173 | 475 | 433 | 399 | 311 | 242.97 | 153.97 | 142.44 | 96.73 | 7.92 | 7.27 | 7.15 | 6.60 |
| **MelSat43-65** | 65 | 320 | 462 | 455 | 205 | 435.65 | 437.25 | 432.33 | 169.70 | 8.77 | 8.77 | 8.76 | 7.41 |
| **MelSat44-96** | 96 | 5 | 14 | 45 | 23 | 4.61 | 8.97 | 28.95 | 12.89 | 2.20 | 3.17 | 4.86 | 3.69 |
| **MelSat45-146** | 146 | 258 | 450 | 253 | 134 | 156.38 | 189.61 | 107.02 | 49.39 | 7.29 | 7.57 | 6.74 | 5.63 |
| **MelSat46-84** | 84 | 27 | 27 | 75 | 78 | 28.44 | 19.77 | 55.14 | 49.96 | 4.83 | 4.31 | 5.79 | 5.64 |
| **MelSat47-240** | 240 | 18831 | 24040 | 383410 | 243120 | 6943.26 | 6162.09 | 98665.74 | 54507.16 | 12.76 | 12.59 | 16.59 | 15.73 |
| **MelSat48-90** | 90 | 37 | 29 | 7 | 35 | 36.38 | 19.82 | 4.80 | 20.93 | 5.19 | 4.31 | 2.26 | 4.39 |
| **MelSat49-103** | 103 | 649 | 1011 | 1209 | 386 | 557.58 | 603.84 | 724.94 | 201.65 | 9.12 | 9.24 | 9.50 | 7.66 |
| **MelSat50-109** | 109 | 7 | 7 | 29 | 5 | 5.68 | 3.95 | 16.43 | 2.47 | 2.51 | 1.98 | 4.04 | 1.30 |
| **MelSat51-80** | 80 | 5 | 3 | 16 | 44 | 5.53 | 2.31 | 12.35 | 29.59 | 2.47 | 1.21 | 3.63 | 4.89 |
| **MelSat52-97** | 97 | 15 | 19 | 31 | 75 | 13.68 | 12.05 | 19.74 | 41.60 | 3.77 | 3.59 | 4.30 | 5.38 |
| **MelSat53-85** | 85 | 17 | 30 | 13 | 105 | 17.70 | 21.71 | 9.45 | 66.47 | 4.15 | 4.44 | 3.24 | 6.05 |
| **MelSat54-70** | 70 | 27144 | 44420 | 1080 | 1456 | 34314.46 | 39037.79 | 952.88 | 1119.20 | 15.07 | 15.25 | 9.90 | 10.13 |
| **MelSat55-69** | 69 | 3 | 11 | 41 | 31 | 3.85 | 9.81 | 36.70 | 24.17 | 1.94 | 3.29 | 5.20 | 4.60 |
| **MelSat56-62** | 62 | 2307 | 4141 | 7314 | 10432 | 3292.74 | 4108.83 | 7285.80 | 9053.57 | 11.69 | 12.00 | 12.83 | 13.14 |
| **MelSat57-76** | 76 | 273 | 242 |  | 7 | 317.87 | 195.89 |  | 4.96 | 8.31 | 7.61 |  | 2.31 |
| **MelSat58-56** | 56 | 571 | 537 |  | 3 | 902.30 | 589.92 |  | 2.88 | 9.82 | 9.20 |  | 1.53 |
| **MelSat59-65** | 65 | 48 | 47 |  | 6 | 65.35 | 44.48 |  | 4.97 | 6.03 | 5.48 |  | 2.31 |
| **MelSat60-172** | 172 |  |  | 16891 | 6119 |  |  | 6065.14 | 1914.24 |  |  | 12.57 | 10.90 |
| **MelSat61-172** | 172 |  |  | 540 | 1936 |  |  | 193.90 | 605.65 |  |  | 7.60 | 9.24 |
| **MelSat62-160** | 160 |  |  | 1292 | 750 |  |  | 498.72 | 252.22 |  |  | 8.96 | 7.98 |
| **MelSat63-60** | 60 |  |  | 1164 | 463 |  |  | 1198.16 | 415.22 |  |  | 10.23 | 8.70 |
| **MelSat64-14** | 14 |  |  | 0 | 0 |  |  | 289841.52 | 62446.67 |  |  | 18.14 | 15.93 |
| **MelSat65-170** | 170 |  |  | 7722 | 5312 |  |  | 2805.40 | 1681.33 |  |  | 11.45 | 10.72 |
| **MelSat66-159** | 159 |  |  | 97 | 99 |  |  | 37.68 | 33.50 |  |  | 5.24 | 5.07 |
| **MelSat67-85** | 85 |  |  | 90 | 79 |  |  | 65.39 | 50.01 |  |  | 6.03 | 5.64 |
| **MelSat68-173** | 173 |  |  | 211 | 61 |  |  | 75.33 | 18.97 |  |  | 6.24 | 4.25 |
| **MelSat69-54** | 54 |  |  | 83 | 114 |  |  | 94.93 | 113.59 |  |  | 6.57 | 6.83 |
| **MelSat70-83** | 83 |  |  | 1035 | 745 |  |  | 770.15 | 482.97 |  |  | 9.59 | 8.92 |
| **MelSat71-95** | 95 |  |  | 60 | 121 |  |  | 39.01 | 68.53 |  |  | 5.29 | 6.10 |
| **MelSat72-173** | 173 |  |  | 390 | 351 |  |  | 139.23 | 109.17 |  |  | 7.12 | 6.77 |
| **MelSat73-76** | 76 |  |  | 2883 | 2338 |  |  | 2342.85 | 1655.29 |  |  | 11.19 | 10.69 |
| **MelSat74-97** | 97 |  |  | 102 | 32 |  |  | 64.94 | 17.75 |  |  | 6.02 | 4.15 |
| **MelSat75-137** | 137 |  |  | 947 | 691 |  |  | 426.92 | 271.39 |  |  | 8.74 | 8.08 |
| **MelSat76-170** | 170 |  |  | 6961 | 2832 |  |  | 2528.93 | 896.37 |  |  | 11.30 | 9.81 |
| **MelSat77-20** | 20 |  |  | 0 | 0 |  |  | 29005.43 | 0.00 |  |  | 14.82 | 0.00 |
| **MelSat78-97** | 97 |  |  | 32 | 19 |  |  | 20.37 | 10.54 |  |  | 4.35 | 3.40 |
| **MelSat79-78** | 78 |  |  | 115 | 49 |  |  | 91.06 | 33.80 |  |  | 6.51 | 5.08 |
| **MelSat80-57** | 57 |  |  | 96 | 49 |  |  | 104.02 | 46.26 |  |  | 6.70 | 5.53 |
| **MelSat81-83** | 83 | 210 | 210 |  |  | 223.89 | 155.65 |  |  | 7.81 | 7.28 |  |  |
| **MelSat82-179** | 179 |  |  | 339 |  |  |  | 116.97 |  |  |  | 6.87 |  |
| **MelSat83-173** | 173 |  |  |  | 1918 |  |  |  | 596.55 |  |  |  | 9.22 |
| **library size** |  | 11300525 | 16255313 | 16191453 | 18584715 |  |  |  |  |  |  |  |  |

**Table S4**. Expression analyses of 4 house-keeping genes in *M. incognita* based on previously validated candidates [65]. For mapping of two juvenile (J2) transcriptome databases (ERR790027 and ERR790028), coding sequences (CDS) of reference genes downloaded from WormBase ParaSite (https://parasite.wormbase.org/index.html) were used as listed *M. incognita* loci for each gene.

|  |  |  |  | **Minc_27** | | | **Minc_28** | | |
| --- | --- | --- | --- | --- | --- | --- | --- | --- | --- |
| **Gene symbol** | **Annotation** | ***M. incognita* loci** | **Length** | **Mappings** | **RPKM** | **log2RPKM** | **Mappings** | **RPKM** | **log2RPKM** |
| *Disu* | protein disulfide-isomerase | Minc3s01268g22290 | 1584 | 2705 | 151.12 | 7.24 | 3035 | 117.87 | 6.88 |
|  |  | Minc3s02350g29607 | 1584 | 2712 | 151.51 | 7.24 | 3041 | 118.10 | 6.88 |
|  |  | Minc3s04154g35616 | 1584 | 2647 | 147.88 | 7.21 | 2938 | 114.10 | 6.83 |
| *Poly* | Polyadenylate-binding protein | Minc3s01682g25559 | 1914 | 2392 | 110.59 | 6.79 | 4797 | 154.18 | 7.27 |
|  |  | Minc3s03281g33396 | 1914 | 2389 | 110.45 | 6.79 | 4790 | 153.96 | 7.27 |
| *ELF* | Elongation factor 2 | Minc3s00468g12850 | 2559 | 1397 | 48.31 | 5.59 | 2155 | 51.81 | 5.70 |
|  |  | Minc3s00594g14836 | 1851 | 838 | 40.06 | 5.32 | 1202 | 39.95 | 5.32 |
|  |  | Minc3s01848g26725 | 1362 | 836 | 54.32 | 5.76 | 1520 | 68.65 | 6.10 |
|  |  | Minc3s02628g30935 | 2559 | 1386 | 47.93 | 5.58 | 2128 | 51.16 | 5.68 |
| *PTP* | protein transport | Minc3s00073g03598 | 1425 | 652 | 40.49 | 5.34 | 908 | 39.20 | 5.29 |
|  |  | Minc3s00981g19546 | 1425 | 629 | 39.06 | 5.29 | 869 | 37.52 | 5.23 |
|  |  | Minc3s03830g34926 | 1425 | 657 | 40.80 | 5.35 | 904 | 39.03 | 5.29 |

**Table S5.**  Consensus sequences of 83 satDNAs found in the genomes of the *Meloidogyne* species by RepeatExplorer.

| **SatDNA** | **Monomer length** | **Consensus sequence** |
| --- | --- | --- |
| MelSat01 | 295 | TTCAATAGACATTTTGACATTTTATTTTTCCAATTCCTTGGTTTAATTACCCAAGTTTAAGGTATGTAAATCATAACTACTTGGGAAAAATTTTGGAATTGTATTTCGAAAGAAATACTTAAAATTAAATCAATAATTAAAAGTTTTTAAAAAAACTTTATCAAAATTTTTAAATTATACTTTTTTTCATAAAAGTATTTTATAGATTATAGAAGGAGAAAAGAAGCAGAGAATGTACACTATCTCTCATTAATTGAAAAAAAAGAAATTTTATTTCCCATCAATCTTTTCCTTA |
| MelSat02 | 174 | CTTATCAGATTTTTTTTCACTGATAAACGAATTTTTGAAATTTCAAATTCCCAAAAAATACACTTTAGTGACTAAATATGGTATTGTTACTCTACAGTAAGTCGATGAAAAAATCTTAACCGGTAATTTTTAAATACAGTTTTTAATGCTGATTCGAATGATATACTATTTATC |
| MelSat03 | 87 | CTTAGTGTATTTTGGTCAGTTATTATGTTCATTATTTTATGATTTATTTTTAACCTATGAATGTAAACTAACCGAATAAAATAAGAC |
| MelSat04 | 89 | TATTCTTTTTTATATAATTAGTCATAATTGAAAATAAGTTTGATAAGAAAATAAAAGTTATTAGGTTTTGGCCTCCAAGTTTATTTTTA |
| MelSat05 | 183 | ATAAATAATCATAAAAAAGAAATAAGTCCCTCTTAAATTTTGGTAATAGGGAATAACCGTTTGTAGACCAATATTTTCTATTCGGTCGTTTTAAAATAAATAAGAATTAAAAACGTCTTAGAATTTATGAAACATTATTAGATTGATAGTTTCAATTCGGTCAACTTATAGGGTCGAATAACG |
| MelSat06 | 103 | TGATTTTCAATACAAATCTTATCAGTCAGAAAATATATAAAGTTACATAATAGAGATCACGAGTTATAGATTCTTATTTTCTTATTATTTATTTCATATCTGT |
| MelSat07 | 80 | TACTTCTTAGTTAATAAGAGAATCAAAAATATCTTAATAGATTTAAGAAATATTCGGTAACATTATTTTTAGACCTCGGT |
| MelSat08 | 134 | TTCAAAATTATTAGTGCCATATTTATTCTAGTAATGTAATCGAATTCGATATAAAAGTTCTACGAACTAAATAAACAGATAATGTCAATTGTGTGTGAATAATAAAAATCTAAATTTTGTTTTCATTTAGACTC |
| MelSat09 | 94 | TTTTTTATTCGAATATATTCGACAAATAAAAGTTTAAACTAATATACGACCCAGTGAAATGCCGGAATTCTTAGACAAAATCGAAGTACTTTTA |
| MelSat10 | 63 | AAAAATATATGTCCTAGTTAAACGCTAACTTCTAGACCTTTTGTACTTTAATATATCCGAATT |
| MelSat11 | 83 | GTGTCTAAGAGTAGGGTTTTAAATTTTATAGAAGTTCTATCCAAAAAATTTCTATTCGGTAGATTGCAAAACGCTCAAATCAA |
| MelSat12 | 74 | GAAGATATCAGAAATTATTTATAACTAATGAGTTATAATAGAATATATTTGTCTAAATAAATCTCGAACTGGCA |
| MelSat13 | 86 | GTATCGAAATTCTTTTTTGTGTTCGTGTATAATTCTCAAATATAATAATAGAAACGAAAAATTTTTGGAAAACAGTATTTGTATTA |
| MelSat14 | 95 | TAAGATATCCTTAAAAATTATAAAAATCTAAACACATATAATAGAATAACGATTAATAAATGAAAAAAATGACAAAGAAAGAAATATAATGTAAA |
| MelSat15 | 162 | TGATTGACGGCATTTTTTTATATACATTTTATTTCTATTATATTTTTTTAATTTTCAAGATTAATTCATAAATGATATAAAACTCAGCATTTTCAGGCTTACTAAAACTGAACATCAATTTTAAATAAATAAAATAAGATTCATAAAATTACACTTCAATTG |
| MelSat16 | 76 | GATTTATATATCCAGAAACTTAGATTCTCTTCGGAATTTTGAGAATAATACTATTTTAATCAGGCTTAGTTTTGTA |
| MelSat17 | 233 | TGTCAGGGGCGTGTCAGAAAAGAAGGGGGTCTACGTCTAATCAAAGGTCTAAGTGCCCTACTGTCCGTACCTATGGAAGACCATCCCGTACCTTGGTATATGTAACCGGGAGGTATGAAAGCCCGATCCTATTGAAATATTATTTTGAAAAAAGGGGGTTGTCAGTTCAGTGCGGAATTTTCAGTGTGGGTGTCATGGACAATTGATCACTGGACATTTGGTCAGTGACACTC |
| MelSat18 | 88 | TTCGGAATCTAAGATTTTAGAAGTAAAAATACAGAGTATTGATTTATTTTTAGATTTTAAATAATCAAAGATATAAAATCAAAAAATT |
| MelSat19 | 175 | TCACAAAAATTATTTTTCGTCATTTTTATTTTAGGTAAGTTAGGATGGAACAAAGTTCTAAACGTGATTAAATACTACAGAAGATGGGCTTTCAAATGAGATATTAAGTATCGTTATCGCACATAAATTCGCGAACCCCGATCCCTTTAAACTTTCCAAGTATGAAATTTTTAAA |
| MelSat20 | 69 | ATTAAAATAATGTCGTATAATATAAAATAATTTCCTGAAACGAACCTAATATAATATTATTAGAGAAAT |
| MelSat21 | 55 | TTGACAGATGTTATTCTTATTATTCCCCTTTATATTTTAATAGGTTCGGGTCGTA |
| MelSat22 | 186 | GGTTCTGCTGCTTTTTGTTTTGCTGCCTCGGCTTCAGCCTTCTGCCTGGCTTCAGTTTCAGCCTTCTTTTTAGCATCATCTAACTTTTGCTTCTCTTGCTTCTGTTTTGCTTCAAGTTCAGCTTTTTGTTTTGCCTCAGCATCGGCTTTCTGCTTTGTTTCTAATTCAGCTTTTTTCTTTGCTTCA |
| MelSat23 | 204 | ATAATATTATTTAATTTCCAAAAATACAAATCGAGTTTTTATCTTTATTATTTTCATTTTATGGGGATATTATACCCAAATAAAGCACATAAATCCCGTTTATATTTCTGAGGTTTATAATTTATTATGCTTAATTATCTGAATATTACCTTCCCTATTAAATAATTTTTAAAAATTATTTTCATATTATTTAAATAATGGAAC |
| MelSat24 | 104 | TATATGATGATCAAACCTATTATATTCTTTTATAATCAACTAATTCATTTATAAAATAATCAGTTATTATTTGACTAATAATAGCGTTTTCTCACTTTCTCTAA |
| MelSat25 | 69 | ATCATATGGGACTCTAGAGTCTCTTCTGAGACTTGAAAATCCAAAAATTTATTTCATTACTGAAATATT |
| MelSat26 | 63 | AATTGACTAAAGCCAAAATAACGGCTACCTAATTAGTCACTAAATCTTTCCGAATACAAACTA |
| MelSat27 | 71 | TAAAATAATTTGAATATGCAAAATTTTAAATCGAAAATCGAAATTTAAATCGAAAATAATTATTTAGAAGA |
| MelSat28 | 54 | TAATAATATTTTCTAAATGGCAAATCTGACATCCAAAATTTGAAAAATAATTAA |
| MelSat29 | 123 | TTTTTAAATGTATTGTTAAATGTATTTTTAAAAAATTGTATTCTTAAATGTATTTTTAAAAATAATTGTATTTTTAAATGTATTTTTAAAAATTAATTGTATTTTTTAAAATAATTATTTTAT |
| MelSat30 | 48 | TATAAATCTTTTGAACGAAATTTTGAATTTCTTCGAAGAAATTTCAAT |
| MelSat31 | 57 | GAATACCAGTAATAGGTCTAAATTCGACCTCCAAATGTAGGCTCTTTGCAAGATGCC |
| MelSat32 | 158 | TTTAAAAAAATTTATAAAATTTCAGAACTTATGGACATCAATCTCATACTCAAAAATTTTGAAAAATCGAAGTATTCTGAAATGTTTTTATAAATTCTTGGGACCAATGGTCCACTGATTATTTCTATAACCGATTTGTACCATAACTTCCGGAATTT |
| MelSat33 | 57 | TCGAATTTCGACAGATTATTGGAATTCGGCTACTTTCAAAAAGCAAATAATTGAAGG |
| MelSat34 | 97 | TAAAAAATACATTGATACAGAAATTTTAGTGAATTATTATGGGAATAATTATTTCGGAACTTAGGAATTTTATATTTATTTTTTGTAATCTTCAGAA |
| MelSat35 | 64 | TTATTCTAAAGTTTACCGCTTAAATAATCCGGAAATGTGAGTGCTTTTGTTAGTTGTTTGGATC |
| MelSat36 | 192 | TTTTCAAGATTTTTACAAATTTTAGAAAATTTGGTCAAAATTGCCGGTCGTTTTTTGATAGAGTATTTCATGCTCTTTCGAATGAGCTATAGAACATTCAAATCAAATTTTAATTCTGGGTGAATTAACACTATAAACTTCGCAGTACCAAAAATTATCCCATAATATACCAAAAATTACCAGTCATAAAAT |
| MelSat37 | 157 | AATTTTAAAATTAAAAAACGAAAAATAATAGTTTATCACTAATAATGTTAATTTCCGTCTTTAAATTTTAGAATTTCTTCGAAAAAATTTTACTACAAAAAAATTTCAGAAAAAATAAAAATGTTCTAAAAATTTAGGGAAAAAATTATTCCGAAGA |
| MelSat38 | 86 | AATATTTTATCTTCTAAAGTTTAAATAAAGTCTGATTTGCCCTAGATTAGTAAATTATTAGTACTAACCATTATTTCAAATATACG |
| MelSat39 | 98 | GAACACATCAGATTTATAGTGCTATATTCTATTATATTCTTTTATAATTCTCAAATAATTTTATATCTCAAACTTGTCTAACGCTCTAAATACGGTTG |
| MelSat40 | 184 | AAAAAATTTGCTGGTAGGCATATTTTATTTAATTTTTTCTTGAAAATTCCTACACGAATTTACACGAACTGGCTAGATCTAGTAGTGACTCGAGCTGCTGCATCCATATCAGGTGAGATCTTCCAGAATTATCGATTTTAAAAAAAGTTATTCCATGGTTAATAAAGAGATAGCCATTTTTTTC |
| MelSat41 | 175 | GCGTTAAAAACTGTATCAAAAAAAGGGAGGTAATTTTTTCTCAATTCAATTTCCAAATAGACTGTACACCAGGAATTAACGTAAGTCGGACATTTTTAATTAAATGAAATATTTGTATAATTTATAATTCACGAATTTAAATTTGAGATAGATGTTCTATATACATTTCGAAAGA |
| MelSat42 | 173 | GGATTCGAATTCGTGATGCCCTGAAGCTTTGAAGTGTATAAGTGCCTAATAATTTTCGATATACTTCATATTTGGCATCACTACTATTGAATAACTTAATGGAAACAAAAAGGCAGAAGGATTCTGATACAGTTTTTTATGCTTGTTCGAATGAGCTATATATCAGCCAAATC |
| MelSat43 | 65 | TCATCTCAATTCTGTTTTTTACAAAAAACTTTTTATTATGGCTCAAAATTTAATCATTATCACTG |
| MelSat44 | 96 | ATTTCTTTTCAATAAAATAAGTTCTAATTAGAAATTAGGTATAACGACCTGTTCTATTAGGTACATTTAGATTCATACGAATATATTGATAAGAAA |
| MelSat45 | 146 | GTTAGTATTTTATCCATTATGACATTTATTTTCAAAGTGGCACAAAAAAGTTATTACAAATTTATTTAAAAAAATTTGACCGAAGATTTTCTAAACTTAATTCAAAGTTGCCGTTGTCAAATAAATTTATTTATTTATGTTTAGCA |
| MelSat46 | 84 | TGTTAGAGATATTCTATTAAAATAAACCAACCGAATAGAAATCAATTTTAGATTTCTCAGAATATTTAATGTTTTCATCACATT |
| MelSat47 | 240 | TCTCTTCTCATCCTCATCTTTCTTCGAATCATCGAGTTTCTTTGGAGGCTCTTCTTTTACGTCTTTTTTCTTAGCTTCCTCATCCTTCTTCTTAGCCAATAATTCATCTCTCATCTTCTTAGCTTCATCTTCCTTCTTTCTCTTATCTTCATCTTCCTTTAATTTTTTCTCTTCCGCCAGTTTTTTGGCTTTTTCCTTATCTTCAGCTTCACGTTGTTTCTTTAAGGCTTCTTCCTTTTT |
| MelSat48 | 90 | AATCCAGATTATTAAGGAACCCTATCCCATATTTAGATTGGATTATTTAAATAGTTATACGAATTTTATTCGTTAATTTCCAAAGTATAA |
| MelSat49 | 103 | TAAAATAAAAGCAATAATAGAAAAATGTGAAAAAGAAAAATATTGTCGAAAACTTCTTATTCCTAAATCTAAATTTCAATTTTCTTCGGTAAAGTTATTCTAC |
| MelSat50 | 109 | TAAATAAATAGCAAAAGATTAAAAAGACTGCTAAAGTCGAAGAATATTTCTGACTAATTCTAAATTTTATATTTTATTCGGTATAATTATTCTACTAAAATAAGTCTAA |
| MelSat51 | 80 | ATTTCTATTATAGCTCTTTATATTTTTATCAATTATTATTTTACATAATCGACAACATATTGAAATAGTTTATAATGATT |
| MelSat52 | 97 | GAAGATGCTTCAGATAAGAAATTTTCTGACTAATTCTAAAATTTAAATTTTATTCCATCGTTTTATTTAAATATAATAATTGCATAAGATTCTGAAA |
| MelSat53 | 85 | TGGCAGGATATTTAGTGTTTTTATCACATTTGTTAGAAATATCTTATTAAAATAAACTCCTCGAATAAAAATCAAAATTTAGAGT |
| MelSat54 | 70 | ATTCTAACTCATTACATGTTCATTGATTGGGTTTTCTAACACATTTTTTCTCTTTATTTACATTTTTATA |
| MelSat55 | 69 | CTTAGAATAAAAATTGTGCTTGTTAAAATAAATAGAAATATATCTATACGTATTTTATGAAAATAATAT |
| MelSat56 | 62 | AATTTACTTCATTTGGGATTCTTCTTCCATTTAGTCTTCTCTTTTTTCTTCCACTCCTTTAA |
| MelSat57 | 76 | GCTAAATTTGGATAATTTTATTTTTATCAGAATATCGAATAAATTTTACAAAAATAATACTAAAAAAAGTTTTTGA |
| MelSat58 | 56 | GGTACTCTCGAAAACAATAGTTTTTTAAAAACCATAATTGGTTAGAGAGGGATAAT |
| MelSat59 | 65 | TATCAAAGTTTAGAAGAAATTTTACAATAATAATACTAAAAATTTTATTTTTATATTTTTATTCT |
| MelSat60 | 172 | TGTTTCAAACACCGTTTGATTTGTCTTCGTATCTTTTATCACCATCGAGAAAATCCATTTTAAATATAATCAGTTAAAAAATAATTACCCCACAAAAGTTTATCGTATTATATCTTATGGTATATTGATCGGGTACAATTCGTCTTGGTATATTATATACAATTATAAGTGC |
| MelSat61 | 172 | AAATTTTCGATTATAACTTTTGTTTAACCAATAGTAGATTAAAAGGCACAAGGTTTCTGATACAGTTTTTAATGCTGATTCTAACGAGCTATTAATAATCCAAAACGGATTTGAATTCGTGATGCCCCATCACTTTAAAGTTTTTAAGTATCAATTAATATCCGATATTTTT |
| MelSat62 | 160 | AAAATGTAGTTGAACCACCTTGGAACTTGTACTCAATTGACCGAATTTAAACGAAATTTTTAAAAAGTTGGAACCATGGGAAATCATGGTTATATTTGAACAAGGACTGCCAGTCCTTGTTAGAATATACAATTAGTTTCAAGTTTCTATAAAGTTTTTG |
| MelSat63 | 60 | AGCCGTTTTATACGCTCAAATTTTATTCTAAATTTTAAAGAAAATAAAGAAATATGAATA |
| MelSat64 | 14 | GATTATTTGTCCCT |
| MelSat65 | 170 | TTTTTTTTTAAATTGAATATTTTTAAATTTGATTTTCTGGATGGTGATACGTGTGCATCTAGTATTGTATATACCATAAGAATCAGCATTAAAAGATGTATCTAAAATACAACCACGACCTGTACCCGATCAATATACAATAAGATATAATCCAATATATAATTTTTTAA |
| MelSat66 | 159 | TTTTTTAATTATTTTTGAAAAATTTTGACCTGTTGTTGCTTCCAGGCCTAATAACTCGAGAAGTATTGATTGTTTTTTCAAAATTTAAAAAGAAATTGTAGGGACTAATCTTGTCCCTACTATTTCTGCAATTTATTCCATTAAATTGCGAAAAAAATT |
| MelSat67 | 85 | ATCCAAAATAAGAGTAAAAACTCTTATAATTATTAGTTTTATATAGAATTATGTAAACTATTCGGTCAATTTAATTATAAATAAC |
| MelSat68 | 173 | CATTAAAAACAGTATCTGAATCCATAAGGTAATTTTATCCAATTTGGCTTAGCTAATACATAGAACCGTGGCAATAAGTATATTGTCTATTTTATGGTACACTGATAATTTTTTGAGTTAAAAATCAATGAGATCAAATTTAATCAGAATTTTGTATGGATTATTCGAAAGAG |
| MelSat69 | 54 | TTCTGGGACTTAACATTATTATGTTTAAGTCCCAGTCTATAAAGAACGTTTTTA |
| MelSat70 | 83 | TAATTAGTTTCGACCCACTTTTAGCCCATCGATTTCATAGTTTTGCAATCTTCATAATAGAAAATTTTTGTATAGAACTTTTA |
| MelSat71 | 95 | AATTTTATGGTAATCAATAATTCTCTATTTCTATTCTTGGACATTTATACAATTTATTCGATAAATTTGACCGAATAGCTTCTAAAACGAGTAAA |
| MelSat72 | 173 | TCGAAATCTTTAGTAAAAGGGCTCGAAATGAAACGAGTCACTGGTATTATATACAGTATTTAATGCTCTTTCGAATGAGATACTGCAGATCCATGTGCGATTCGATACCACAATTCGCACTCGGGTTGAATACCCCATGTCCCAAAAAATAAAATATTTATTTTATTTTCGAC |
| MelSat73 | 76 | GCATGAAAAACACAAAAATACAAAAAATAAAAAAACACAAAAAAATTCAATACTATTAAAATGTGAGTTGCCAAAA |
| MelSat74 | 97 | CTATTACATTTATTTCAAAAAGTTTCAGGTTTTTAAAAAATAAGAATAGAACCTGAAACACTATTGTCAAAAACGTGGAAGGATCTATTATCACAAA |
| MelSat75 | 137 | TGAATTTTTCAAATTTAACTTAATATAACGGATCCAATATTGTTTTTATTTTTTATCTTTAGGTTAGTTTTTTTACTTCTAATTTTTGAGTTTTCCAAAAAATTATTTTATAGGTTTTCCATTTATGTGATTTTTTT |
| MelSat76 | 170 | GGATATGTGCGACTGGAGGTTTCGATATACCATTCGAATCTCCATTAAATATAGTATCTAATTTACCAAGACGAATTGTACCCGATTAATATACCGTAAGATATTTATGGATTAATAATTTTGGGTTATTTTTTTTGACTGACAATCTTCAAAGTGGATTTCCTCGATGA |
| MelSat77 | 20 | CCAAGATGTTCCAGAGTGTT |
| MelSat78 | 97 | ACAATATATTCTTTTTCAAATTAATTATGTTTGTTTTGATTAGCTCAGTACGTATTGTTCGGGATTGTACTCAATTATTGATCATTATTTTTATAAA |
| MelSat79 | 78 | CTGCTTTTTGACAATAGATTGTCAGGTTGTATTCTGATTTTTTAAAAACATGATACTTTTTTGAATAATACAACCTTA |
| MelSat80 | 57 | GTCAACTTTAAATTAACCTATAAATATTGGACGAATTTAGTCCTGTTATTGGAATTC |
| MelSat81 | 83 | CTAAAAACTGTGAAAAATAACCAGTAATGCACATAAATAAGGATTTATTTAGAATTTTCGTTCATATTATTGATGTTTACAGT |
| MelSat82 | 179 | TTTCATTTAATTTAAGATAACTCGGGAACTAAACAAAAATATAACTCACGAAATCTCTATATTATTTGGCAATTAATGTGCTCAAAAATCCTCAGTCAAATTATTTTATTTCATTCTCTATCAATAATTATTTATTATTAATCTTATCTGATATATCTTGTTTAGATTCTTTTAACGAG |
| MelSat83 | 173 | GAAGCAATGCCAAATATGAAAAATATCAGATATTATTTTGCACTCATAGACTTCAAAGCATTGTGGCATCACGAATTCAAATCAAATTTAGTTTAACAATAGCTCATTCGAACGAGCATTAAATACTCTATCAGAATCCTTGTGCCTTTCATTTAACTATTAGTTATTCAAAA |

**Table S6.** Primers pairs used for MelSat amplification and labeling.

| SatDNA | Forward primer | Reverse primer |
| --- | --- | --- |
| MelSat01 | CTCTGCCCAATGAGCTGTCC | CTCTGCCCTCACATTAAG |
| MelSat02 | TTACTCTACAGTAAGTCGATG | CAATACCATATTTAGTCAC |
| MelSat36 | TTGGTCAAAATTGCCGGTCG | GAAGTTTATAGTGTTAATTCACCC |
| MelSat42 | GGAAACAAAAAGGCAGAAGG | AGTTATTCAATAGTAGTGATG |
| MelSat61 | AGGCACAAGGTTTCTGATACAG | TGATGGGGCATCACGAATTC |
| MelSat72 | GCAGATCCATGTGCGATTC | TGGGACATGGGGTATTCAAC |
| MelSat83 | AAAGCATTGTGGCATCACG | TGAAAGGCACAAGGATTCTG |
